# β-Microseminoprotein Endows Post Coital Seminal Plasma with Potent Candidacidal Activity by a Calcium- and pH-Dependent Mechanism

**DOI:** 10.1371/journal.ppat.1002625

**Published:** 2012-04-05

**Authors:** Anneli M. L. Edström Hägerwall, Victoria Rydengård, Per Fernlund, Matthias Mörgelin, Maria Baumgarten, Alexander M. Cole, Martin Malmsten, Birthe B. Kragelund, Ole E. Sørensen

**Affiliations:** 1 Division of Infection Medicine, Department of Clinical Sciences Lund, Lund University, Lund, Sweden; 2 Division of Dermatology, Department of Clinical Sciences Lund, Lund University, Lund, Sweden; 3 Division of Clinical Chemistry, Department of Laboratory Medicine Malmö, Lund University, Malmö, Sweden; 4 Burnett School of Biomedical Sciences, University of Central Florida College of Medicine, Orlando, Florida, United States of America; 5 Department of Pharmacy, Uppsala University, Uppsala, Sweden; 6 Structural Biology and NMR Laboratory, Department of Biology, University of Copenhagen, Copenhagen, Denmark; Albert Einstein College of Medicine, United States of America

## Abstract

The innate immune factors controlling *Candida albicans* are mostly unknown. Vulvovaginal candidiasis is common in women and affects approximately 70–75% of all women at least once. Despite the propensity of *Candida* to colonize the vagina, transmission of *Candida albicans* following sexual intercourse is very rare. This prompted us to investigate whether the post coital vaginal milieu contained factors active against *C. albicans*. By CFU assays, we found prominent candidacidal activity of post coital seminal plasma at both neutral and the acid vaginal pH. In contrast, normal seminal plasma did not display candidacidal activity prior to acidification. By antifungal gel overlay assay, one clearing zone corresponding to a protein band was found in both post coital and normal seminal plasma, which was subsequently identified as β-microseminoprotein. At neutral pH, the fungicidal activity of β-microseminoprotein and seminal plasma was inhibited by calcium. By NMR spectroscopy, amino acid residue E_71_ was shown to be critical for the calcium coordination. The acidic vaginal milieu unleashed the fungicidal activity by decreasing the inhibitory effect of calcium. The candidacidal activity of β-microseminoprotein was mapped to a fragment of the C-terminal domain with no structural similarity to other known proteins. A homologous fragment from porcine β-microseminoprotein demonstrated calcium-dependent fungicidal activity in a CFU assay, suggesting this may be a common feature for members of the β-microseminoprotein family. By electron microscopy, β-microseminoprotein was found to cause lysis of *Candida*. Liposome experiments demonstrated that β-microseminoprotein was active towards ergosterol-containing liposomes that mimic fungal membranes, offering an explanation for the selectivity against fungi. These data identify β-microseminoprotein as an important innate immune factor active against *C. albicans* and may help explain the low sexual transmission rate of *Candida*.

## Author Summary

The innate immune factors controlling *Candida albicans* are mostly unknown. Sexual transmission of *Candida* during vaginal intercourse is very rare. This prompted us to investigate whether the post coital vaginal milieu contained innate immune factors active against *Candida*. We found potent candidacidal activity of acidic post coital seminal plasma mediated by β-microseminoprotein, while seminal plasma did not possess any fungicidal activity prior to acidification. The fungicidal effect of β-microseminoprotein was regulated by a novel calcium and pH-dependent mechanism uniquely suited for the post coital vaginal environment. At neutral pH, the fungicidal activity of β-microseminoprotein was inhibited by calcium. The acidic vaginal pH, on the other hand, unleashed the fungicidal activity by decreasing the inhibitory effect of calcium. The fungicidal activity of β-microseminoprotein was mapped to a fragment of the C-terminal domain with no structural similarity to other known proteins. Experiments with a homologous fragment from porcine β-microseminoprotein demonstrating calcium-dependent fungicidal activity suggest this to be a common feature for members of the β-microseminoprotein family. These data may help explain the low transmission rate of *Candida* after vaginal sexual intercourse.

## Introduction

Although innate immunity plays a major role in the host defense against *Candida albicans* infections, the specific innate immune factors controlling *Candida* are unknown [1]. Mucosal infections with *Candida* are common, in particular vulvovaginal candidiasis in women. This condition affects approximately 70–75% of all women once during their lifetime and 5–8% have recurrent vulvovaginal *Candida* infections [2].

While frequent sexual intercourse is associated with bacterial vaginosis, this is not the case with vaginal candidiasis [3] despite the propensity of *Candida* to colonize the vagina. This difference between sexual transmission of bacterial vaginosis and vaginal candidiasis is striking since seminal plasma contains potent antibacterial activity [4], [5], [6], [7] playing a role in limiting bacterial proliferation after sexual intercourse. Indeed, receptive orogenital sexual intercourse carries a higher risk of *Candida* transmission than vaginal intercourse [3] and penile-vaginal transmission is very rare [2]. Despite the fact that human seminal plasma contains potent antibacterial and antiviral activity [4], [6], [7], [8], we found no studies describing significant antifungal activity of seminal plasma or vaginal fluid against *Candida*. This prompted us to investigate whether the postcoital vaginal milieu contained innate immune factors active against *Candida*.

In this study, we found that the post coital seminal plasma contained potent candidacidal activity while no major candidacidal activity was found in normal seminal plasma at neutral pH or in vaginal fluid. Subjection of seminal plasma to the acidic vaginal pH found post coitally endowed seminal plasma with potent candidacidal activity. The candidacidal activity was mediated by β-microseminoprotein (MSP, also named PSP94), a protein with no previously known antimicrobial activity, and regulated by a novel calcium- and pH-dependent mechanism uniquely suited for the post coital vaginal environment. At the neutral pH of seminal plasma, the fungicidal activity of MSP was inhibited by calcium binding. After sexual intercourse the acidic vaginal pH unleashed the fungicidal activity of MSP by a decreased inhibitory effect of calcium. The fungicidal activity of MSP was mapped to a region of MSP with no structural similarity to other known proteins. These data may help explain the low transmission rate of *Candida* after vaginal intercourse.

## Results

### Candidacidal effect of post coital seminal plasma is due to acidification

**Figure 1:**
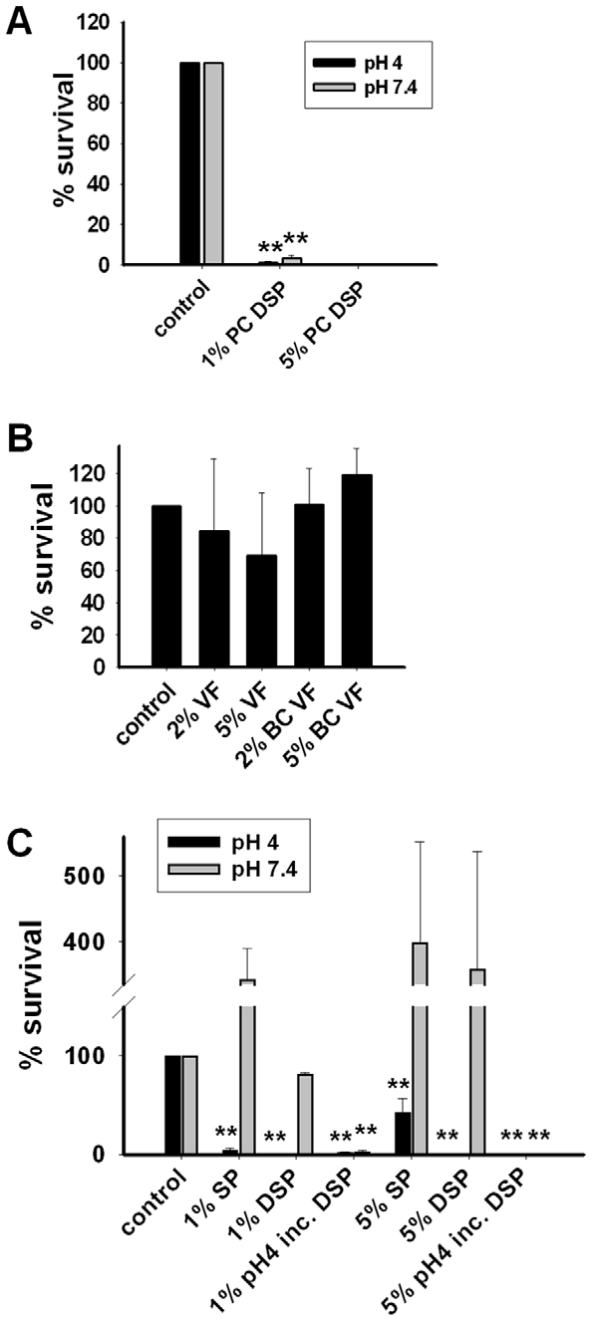
Candidacidal effect of post coital seminal plasma is due to acidification. **A.** Dialyzed post coital seminal plasma (PC SP) (5% and 1%) was tested in CFU assay with *C. albicans* at both pH 4 (black bars) and pH 7.4 (grey bars). Results are shown as average from three independent experiments. Three different post coital samples were examined with similar results. Complete killing was found in the experiments with 5% post coital seminal plasma. Consequently no error bars are shown. All percentages refer to % volume. **B.** Vaginal Fluid (VF) (three donors) and buffer changed vaginal fluid (BC VF) were tested in CFU assay against *C. albicans*. Results are shown as average from three independent experiments. All percentages refer to % volume. **C.** Seminal plasma (SP) and dialyzed seminal plasma (DSP) were tested in a CFU assay with *C. albicans* at pH 4 (black bars) and pH 7.4 (grey bars). Seminal plasma displayed candidacidal activity in CFU assay only at pH 4. Candidacidal activity of seminal plasma was observed in CFU assay at neutral pH when the seminal plasma had been incubated at pH 4 followed by neutralization of pH by dialysis (pH 4 inc. DSP). Results are shown as average from three independent experiments. Complete killing was found in all experiments with 5% pH 4-incubated seminal plasma as well as with 1% DSP at pH 4. Consequently no error bars are shown for these experiments. All percentages refer to % volume. ** denotes p<0.001. Error bars indicate standard deviations. Further details regarding the statistical analysis are found in Dataset S1.

Since *Candida* is rarely transmitted by vaginal intercourse, we investigated the activity of post coital seminal plasma collected as a post coital vaginal aspirate 8–10 hours after sexual intercourse. The post coital seminal plasma had acidic pH (∼4) and was dialyzed to unmask the activity of salt sensitive antimicrobial peptides (AMPs). The post coital seminal plasma displayed prominent candidacidal activity (Figure 1 A) both at neutral and at the vaginal pH (pH 4). Subsequently, antifungal activity of 3 samples of vaginal fluid aspirated the same way as the post coital seminal plasma was investigated (Figure 1 B). To validate the absence of the seminal plasma proteins in the vaginal fluid samples, dot blot was performed for hCAP-18, a cathelicidin present in high concentration in seminal plasma and neutrophils. The hCAP-18 concentration in the vaginal fluid samples was less than 0.5% than that found in seminal plasma (data not shown). Since some neutrophil-derived hCAP-18 probably will be present in vaginal fluid, these data show that seminal plasma proteins were virtually absent from the tested vaginal fluid samples. Compared to post coital seminal plasma, no significant antifungal activity was found for vaginal fluid samples tested in the same dilutions as post coital seminal plasma. Subsequently, we tested the candidacidal activity of normal seminal plasma. At the acidic vaginal pH, normal seminal plasma was just as potently candidacidal as post coital seminal plasma (Figure 1 C). However, no antifungal activity was observed for normal seminal plasma at neutral pH even after extensive dialysis (Figure 1 C). Indeed seminal plasma without fungicidal effect seemed to increase the growth of *C. albicans*. Two samples of seminal plasma collected after 4 hours in the vagina that had neutral pH did not display antifungal activity at neutral pH (data not shown). Both non-dialyzed seminal plasma and dialyzed seminal plasma displayed prominent fungicidal effects at a 1000-fold dilution (0.1%). Further dilutions diminished the fungicidal effect (data not shown). Since the post coital seminal plasma also contained antifungal activity at neutral pH, normal seminal plasma was subjected to the low vaginal pH followed by re-neutralization of the pH by dialysis. After subjection to the vaginal pH, seminal plasma was antifungal even at neutral pH. Subsequent experiments demonstrated that pH 5 or lower endowed seminal plasma with antifungal activity (data not shown).

Apart from *C. albicans* ATCC 90028 used in the experiments above, the antifungal activity of dialyzed seminal plasma was tested in CFU assays against six other *C. albicans* strains, four *C. parapsilosis* strains, and four *C. glabrata* strains (Figure S1). All *C. albicans* and *C. parapsilosis* strains were sensitive to seminal plasma at low pH but not at neutral pH. None of the *C. glabrata* strains were sensitive to seminal plasma even at low pH. In these experiments, seminal plasma often seemed to have a growth stimulatory effect if the *Candida* were not susceptible to killing.

### The fungicidal activity of seminal plasma was mediated by MSP

**Figure 2:**
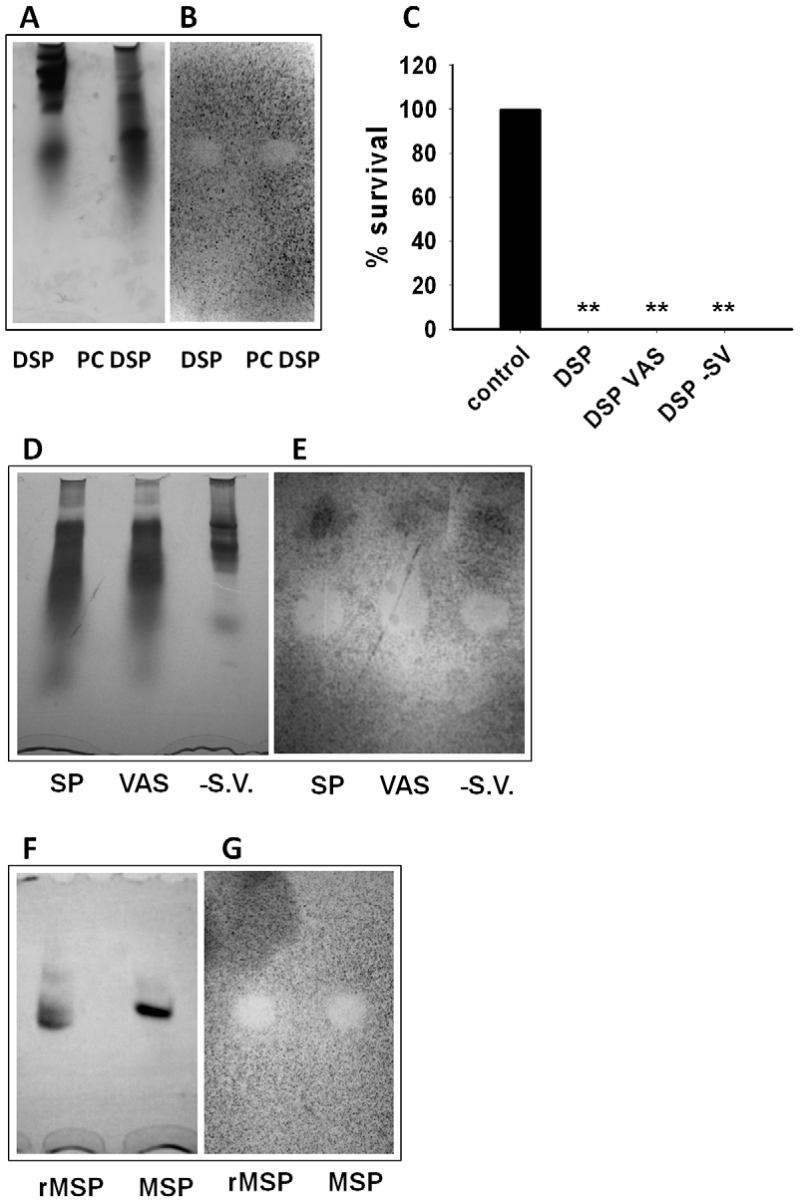
Antifungal gel overlay assays with seminal plasma and MSP. Post coital seminal dialyzed plasma (PC DSP) and seminal plasma (DSP) were analyzed on an AU-PAGE gel and analyzed by coomassie staining (**A**) or in an antifungal gel overlay assay with *C. albicans*. (**B**). Clearing zones depict antifungal activity. The antifungal activity of dialyzed normal seminal plasma (DSP), seminal plasma from a vasectomized patient (DSP VAS), and a patient with deficient seminal vesicles (DSP -SV) were analyzed by CFU assay (**C**). Results are shown as the average of three independent experiments. ** denotes p<0.001. Complete killing was found in the three samples of seminal plasma and, consequently, no error bars are shown. Further details regarding the statistical analysis are found in Dataset S1. Normal seminal plasma (SP), seminal plasma from a vasectomized patient (VAS), and from a patient with deficient seminal vesicles (-SV) were analyzed on an AU-PAGE gel by coomassie staining (**D**) or in an antifungal gel overlay assay with *C. albicans* (**E**). Recombinant MSP (rMSP) or native MSP were analyzed by AU-PAGE gel by coomassie staining (**F**) or in an antifungal gel overlay assay with C. albicans (**G**). MSP gave clearing zones migrating identically to the zones found in seminal plasma. Acid-urea PAGE is a non-detergent based PAGE where the electrophoretic mobility of a protein is determined both by charge and size. Two proteins of similar size can consequently have very different mobility. Accordingly, molecular size standards were not used.

Seminal plasma and post coital seminal plasma were analyzed by AU-PAGE (Figure 2 A) and antifungal activity was tested in a gel overlay assay. One distinct clearing zone was found (Figure 2 B) in both normal seminal plasma and post coital seminal plasma. These clearing zones displayed similar electrophoretic mobility. Together with the CFU assays, this demonstrated that the (poly)peptide(s) responsible for the candidacidal activity both in normal seminal plasma and post coital seminal plasma probably were of the same origin and that the candidacidal (poly)peptide(s) originated from seminal plasma.

To identify the origin of the candidacidal (poly)peptide(s) present in seminal plasma, seminal plasma from vasectomized patients (no contribution from testis and epididymis) and seminal plasma from patients with seminal vesicle dysfunction (no contribution from seminal vesicles) were tested in a CFU assay and in antifungal gel overlay assay. The three seminal plasma samples displayed similar candidacidal activity in the CFU assay (Figure 2 C). In all samples, a single clearing zone with identical electrophoretic mobility was seen in the antifungal gel overlay assay (Figure 2 E). This indicated that the candidacidal (poly)peptide(s) originated either from vas deferens or the prostate.

To identify the candidacidal (poly)peptide(s), a sample of seminal plasma was incubated for 24 hat 37°C to reduce the protein content by degradation of the semenogelins [7]. This sample was analyzed by AU-PAGE and by antifungal gel overlay assay. Control experiments demonstrated that the incubation did not reduce the candidacidal activity in CFU assays. The band on the gel corresponding to the clearing zone was excised and identified by mass spectrometry as MSP in two independent experiments. Subsequently, recombinant MSP as well as native MSP purified from seminal plasma were tested in a gel overlay assay and both displayed antifungal activity by producing a clearing zone with electrophoretic mobility corresponding to the one produced by seminal plasma (Figure 2 G). The two other major proteins from the prostate, prostate specific antigen and prostate acid phosphatase [9], did not give rise to clearing zones in gel overlay assays (data not shown).

### Candidacidal activity of seminal plasma and MSP was inhibited by calcium

**Figure 3:**
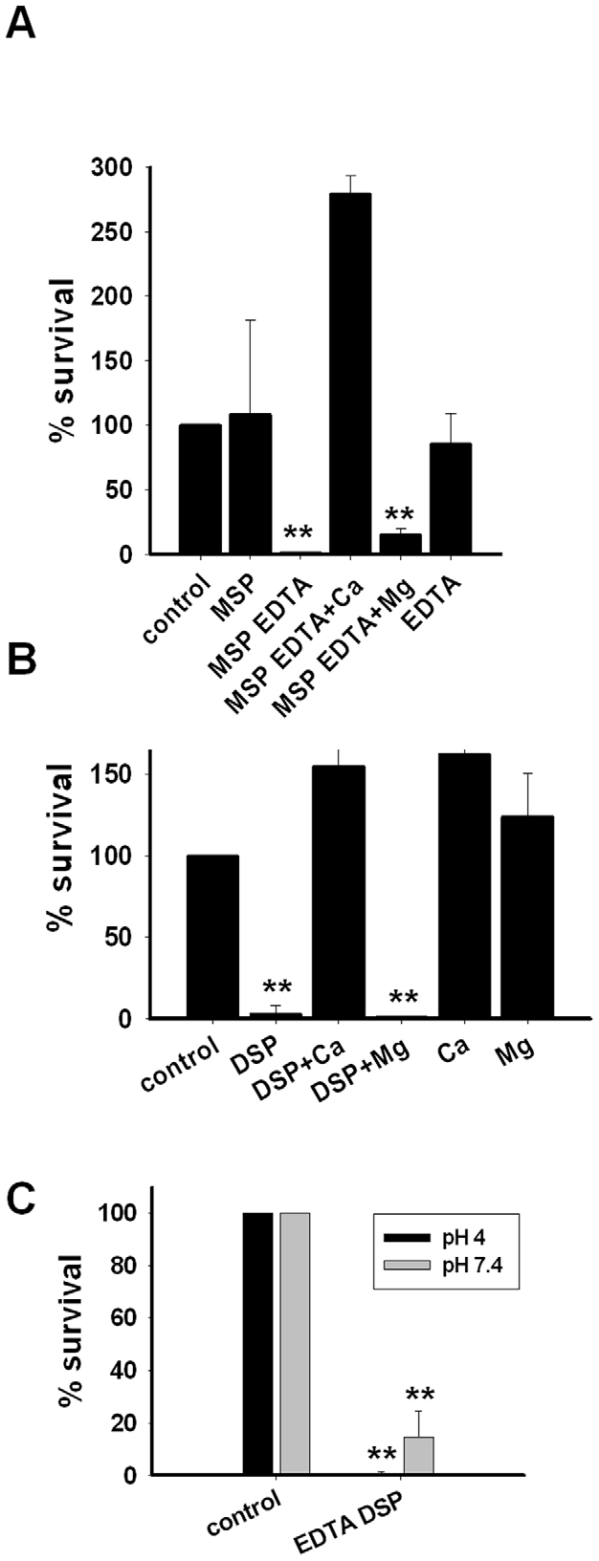
Antifungal activity of seminal plasma and MSP was inhibited by calcium. **A.** Native MSP (25 µM) was tested in CFU assays with *C. albicans* at pH 4. Significant candidacidal activity was seen only after addition of equimolar (25 µM) EDTA. The candidacidal activity was inhibited by 0.5 mM calcium but not 0.5 mM magnesium. EDTA did not display candidacidal activity. **B.** Dialyzed seminal plasma (DSP) (1% volume) was tested in CFU assays at pH 4 in the presence of 0.5 mM CaCl_2_ or 0.5 mM MgCl_2_. The fungicidal activity was inhibited by 0.5 mM calcium but not 0.5 mM magnesium. **C.** Seminal plasma was treated with EDTA followed by dialysis. The EDTA-treated seminal plasma (EDTA DSP) displayed fungicidal activity in CFU assay with *C. albicans* at pH 7.4 without prior subjection to low pH. All results are shown as the average of three independent experiments. ** denotes p<0.001. Error bars indicate standard deviations. Further details regarding the statistical analysis are found in Dataset S1.

To study the pH-dependent mechanism of the MSP-mediated candidacidal activity in seminal plasma, native MSP was tested in a CFU assay with *C. albicans* both at neutral and at acidic pH. Surprisingly, no candidacidal activity was observed even at acidic pH (Figure 3 A). However, addition of equimolar concentration of EDTA endowed MSP with fungicidal activity while EDTA by itself was not fungicidal (Figure 3A), suggesting that divalent cations inhibited the activity of MSP. Addition of 0.5 mM CaCl_2_ abolished the fungicidal effect of EDTA-activated MSP at acidic pH while addition of 0.5 mM MgCl_2_ did not (Figure 3A). Likewise, the candidacidal activity of seminal plasma at pH 4 was abolished by addition of 0.5 mM CaCl_2_ but not of 0.5 mM MgCl_2_ (Figure 3 B). Consequently, normal seminal plasma was treated with EDTA followed by dialysis at neutral pH. The EDTA-treated seminal plasma displayed candidacidal activity in a CFU assay at neutral pH without prior subjection to low pH (Figure 3 C).

### The C-terminus of MSP was responsible for the fungicidal activity

**Figure 4:**
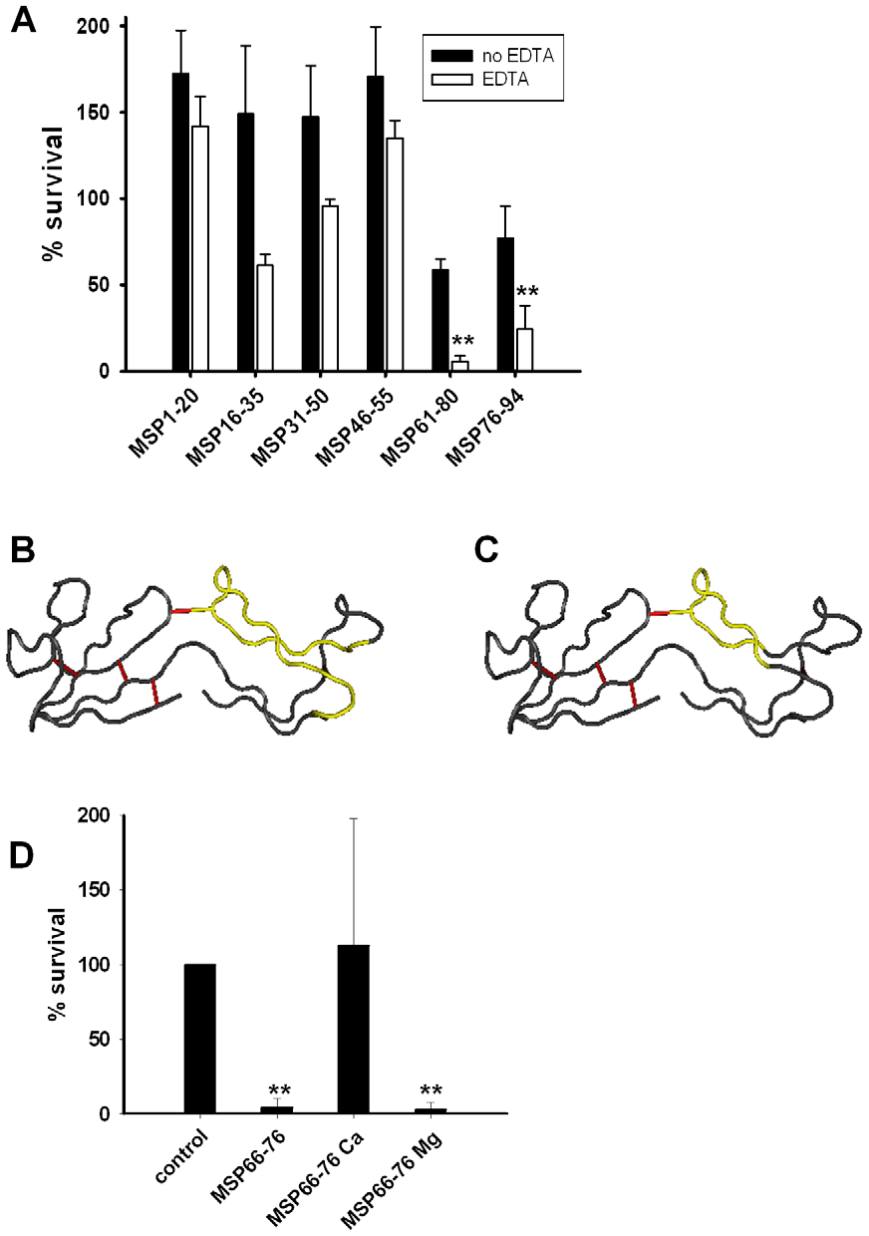
Antifungal activity of MSP-derived peptides. **A.** Six overlapping peptides of 19–20 residues encompassing the entire MSP sequence (in a concentration of 5 µM) were tested in CFU assays at pH 4 against *C. albicans* with (white bars) or without EDTA (black bars). MSP_61–80_ displayed the most prominent fungicidal activity. Results are shown as the average from three independent experiments. **B.** Solution structure of MSP according to Ghasriani, et al [10] with MSP_61–80_ shown in yellow. Disulfide bridges are shown in red. **C.** Solution structure of MSP according to Ghasriani, et al [10] with MSP_66–76_ shown in yellow. Disulfide bridges are shown in red. **D.** MSP_66–76_ (140 µM) was tested in a CFU assay at pH 4. The fungicidal activity was inhibited by 0.5 mM calcium chloride but not 0.5 mM magnesium chloride. All results are shown as the average from three independent experiments. ** denotes p<0.001. Error bars indicate standard deviations. Further details regarding the statistical analysis are found in Dataset S1.

To investigate the mechanism behind the calcium and pH-dependent activity of MSP, we sought to identify the region of MSP responsible for the fungicidal activity. A set of six peptides of 19–20 residues, encompassing the entire MSP sequence with five overlapping amino acid residues (Table 1), was tested in a CFU assay at acidic pH against *C. albicans* (Figure 4 A). MSP_61–80_ (encompassing residues 61–80) displayed the highest fungicidal activity of the tested peptides. Similar to MSP, the fungicidal effect of this peptide was increased by addition of EDTA. The solution structure of MSP has been solved by NMR [10]. MSP_61–80_ forms a positively charged loop that theoretically could be accessible to interact with negatively charged microbial surfaces (Figure 4 B). A smaller version of MSP_61–80_ encompassing the eleven residues at the very tip of the loop (residue 66–76), MSP_66–76_ (Figure 4 C), was tested in a CFU assay and found to be fungicidal. At high concentrations, MSP_66–76_ was fungicidal even without addition of EDTA. As with MSP and seminal plasma, the activity of MSP_66–76_ was inhibited by CaCl_2_ but not by MgCl_2_ (Figure 4 D).

**Table 1:** Amino acid sequence of MSP and MSP-derived peptides.

Amino acid sequence	Peptide
SCYFIPNEGVPGDSTRKCMD	MSP_1–20_
RKCMDLKGNKHPINSEWQTD	MSP_16–35_
EWQTDNCETCTCYETEISCC	MSP_31–50_
EISCCTLVSTPVGYDKDNCQ	MSP_46–65_
KDNCQRIFKKEDCKYIVVEK	MSP_61–80_
IVVEKKDPKKTCSVSEWII	MSP_76–94_
RIFKKEDCKYI	MSP_76–94_
RIFKKQDCKYI	MSP_66–76E/Q_
TNKCQKILNKKTCTYTVVEK	Porcine MSP

The MSP-derived peptides over time lost their fungicidal activity in CFU assays without EDTA even when stored at −20°C. However, the activity was restored by addition of EDTA. The same was observed for the fungicidal activity of acidic pH-activated seminal plasma stored at neutral pH.

### The calcium effect on the antifungal activity was diminished by the acid vaginal pH

**Figure 5:**
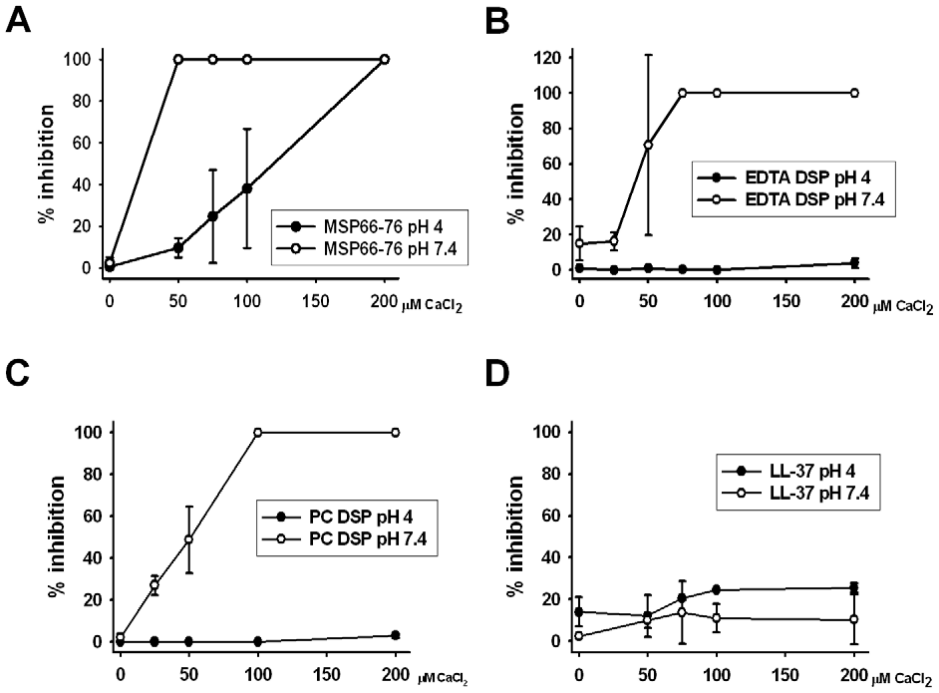
pH-dependent calcium inhibition of fungicidal activity of MSP and seminal plasma. **A.** Inhibition of antifungal activity of 50 µM MSP_66–76_ by calcium. In the experiments at pH 7.4, an equimolar concentration of EDTA (50 µM) had to be added to MSP_66–76_ to observe a fungicidal effect even with no calcium addition. Importantly, no EDTA was added to MSP_66–76_ at pH 4. At pH 4, significant killing of *C. albicans* was found with no calcium (MSP_66–76_) and with 50 µM calcium chloride (p<0.001) and in four out of five experiments with 75 µM calcium chloride. 50 µM calcium chloride completely inhibited killing at pH 7.4. Results are shown from five independent experiments. Error bars indicate standard deviations. **B.** Inhibition of antifungal activity of 5% (volume) EDTA-treated and dialyzed seminal plasma (EDTA DSP) by calcium. At pH 7.4 there was significant antifungal activity (p<0.01) at 25 µM calcium chloride but no significant antifungal activity at 50 µM calcium chloride. At pH 4, there was significant (p<0.01) antifungal activity even at 200 µM calcium chloride. Results are shown from three independent experiments. Error bars indicate standard deviations. **C.** Inhibition of antifungal activity of 5% (volume) post coital dialyzed seminal plasma (PC DSP) by calcium. At pH 7.4 there was significant antifungal activity (p<0.01) at 25 µM calcium chloride but no significant antifungal activity at 50 µM calcium chloride. At pH 4, there was significant (p<0.01) antifungal activity even at 200 µM calcium chloride. Results are shown from three independent experiments. Error bars indicate standard deviations. (**D**) Inhibition of antifungal activity of 50 µM LL-37 by calcium. There was significant antifungal effect of LL-37 in all samples (p>0.01). Results are shown from two independent experiments. Error bars indicate standard deviations. Further details regarding the statistical analysis are found in Dataset S1.

To test whether the pH-dependent mechanism of the antifungal activity of MSP and seminal plasma was due to different effects of calcium at low and neutral pH, antifungal CFU assays were performed at neutral and low pH with MSP_66–76_ and increasing concentrations of calcium. At neutral pH, 50 µM of calcium completely inhibited the antifungal activity of the MSP_66–76_ peptide even when an equimolar concentration of EDTA was present to activate the fungicidal effect. However, at low pH, the antifungal activity of MSP_66–76_ was first completely inhibited at higher calcium concentrations, even without any EDTA (Figure 5 A). To verify the importance of these findings for normal seminal plasma, CFU assays were performed at neutral pH with seminal plasma treated with EDTA and subsequently dialyzed. The fungicidal activity of the EDTA-treated seminal plasma was completely abolished by 100 µM calcium at neutral pH while this was not the case even with 200 µM calcium at low pH (Figure 5 B). The same pattern was found for the post coital seminal plasma (Figure 5 C). Importantly, this pH-dependent inhibition by calcium was not observed when similar experiments were performed with the antimicrobial peptide LL-37 (Figure 5 D). These data demonstrated that the pH-dependent activation of MSP-mediated fungicidal activity was due to decreased calcium inhibition at low pH.

### Calcium inhibited candidacidal activity of MSP_66–76_ via coordination at E_71_

**Figure 6:**
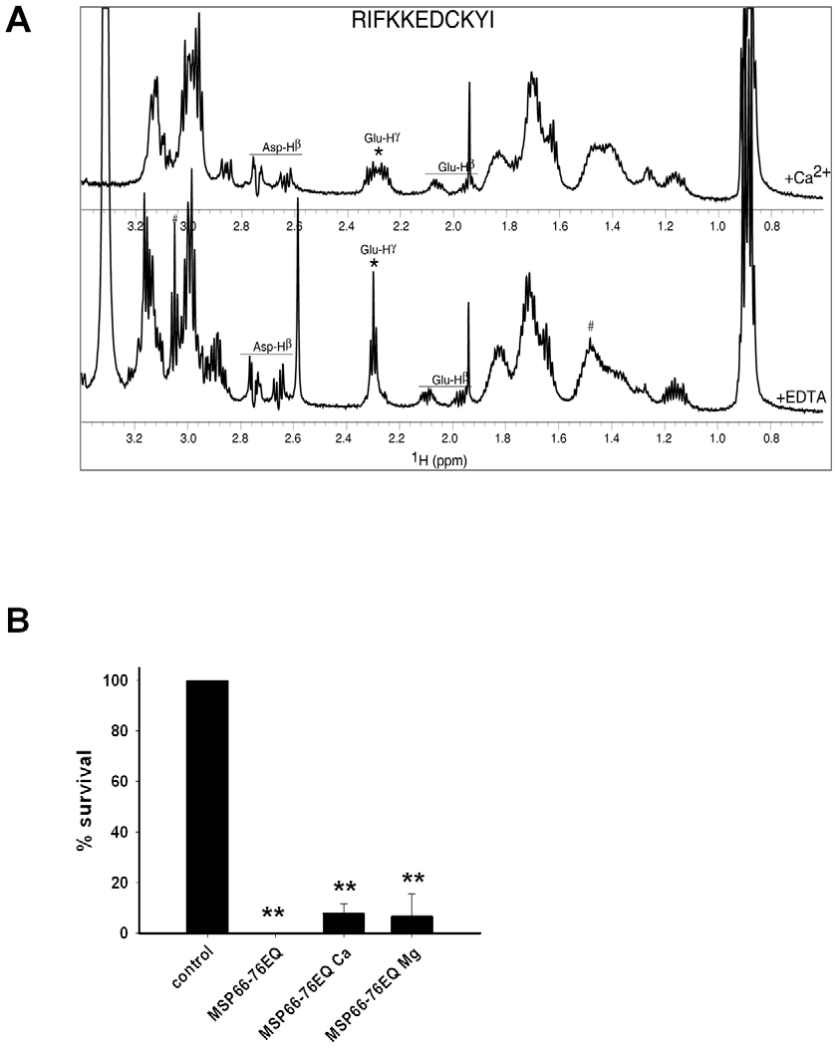
Identification of calcium binding to MSP_66–76_. **A.**1D ^1^H-NMR spectra of MSP_66–76_ in molar excess of CaCl_2_ (upper panel) and EDTA (lower panel) were recorded at pH 7.4. Several signals were affected by the presence of CalCl_2_, most significantly the H^γ^-protons of the single Glu-residue (marked by a star *), where the characteristic triplet in the presence of EDTA is split into several multiplets at 2.28 ppm in the presence of CaCl_2_. Also, signals originating for Lys side chains are affected (marked by #), whereas the signals from Asp H^β^'s are only slightly shifted. **B.** MSP_66–76E/Q_(140 µM), where the glutamic acid residue E_71_ was substituted with a glutamine (Q), was tested in CFU assay at pH 4. The fungicidal activity was neither inhibited by 0.5 mM calcium nor 0.5 mM magnesium. All results are shown as average from three independent experiments.

To investigate whether the inhibitory effect of calcium on the antifungal activity was due to a direct binding of calcium to MSP, MSP_66–76_ was analyzed by CD and NMR-spectroscopy in the presence or absence of calcium. While no significant structural changes were observed by far-UV CD spectroscopy (data not shown), the chemical shifts of the H^γ^-protons of the single glutamic acid residue (E_71_) changed significantly in the presence of calcium as observed by NMR spectroscopy (Figure 6 A). To verify the importance of calcium binding to E_71_, a mutated form of MSP_66–76_ named MSP_66–76E/Q_ was synthesized where E_71_ was substituted with a glutamine (Q). As expected, MSP_66–76E/Q_ had similar antifungal activity as MSP_66–76_ but the antifungal activity was not abolished by calcium at acidic pH (Figure 6 B), thereby demonstrating the location of the calcium-dependent inactivation of MSP. Further data regarding the calcium and pH-dependent candidacidal activity of MSP_66–76_ variants are found in Figure S2.

### Calcium does not influence dimerization of MSP

MSP has been demonstrated to be a monomer at pH 4.5 and a dimer at pH 8 [11]. To investigate whether binding of calcium played a role in dimerization, MSP was analyzed by size exclusion chromatography at pH 7.5 in a buffer containing either calcium or EDTA. In both instances, the protein eluted as a dimer, demonstrating that calcium-binding did not influence dimerization of MSP (Figure S3).

### A peptide from porcine MSP displayed fungicidal activity

Porcine MSP has 50% sequence identity [12] and high homology with human MSP [10]. In order to determine if it also exhibited antifungal activity, a peptide from porcine MSP corresponding to human MSP_61–80_ was synthesized (named PMSP). This peptide had even higher fungicidal activity than its human counterpart (Figure S4), indicating the antifungal activity was present in porcine MSP as well as in human MSP. Even though the residues in the tip of the candidacidal loop in the porcine MSP were a lysine and a threonine (compared to a glutamic acid and aspartic acid in human MSP), the antifungal activity was inhibited by calcium but not magnesium (data not shown).

### MSP did not display antibacterial or cytotoxic activity and selectively caused leakage of fungal-like liposomes

To determine if MSP also had antibacterial activity, CFU assays with *E. coli*, *S. agalactiae*, *S. pyogenes*, *S. aureus* and *E. faecalis* were performed. The assays with *E. coli* and *E. faecalis* were performed at both low and neutral pH, whereas the assays against the other bacteria were performed at neutral pH and EDTA since these bacteria did not survive at pH 4. We found no antibacterial activity against any of the bacterial strains at either low or neutral pH in a CFU assay with purified MSP (10 µM MSP+20 µM EDTA) or with recombinant MSP (10 µM dialyzed MSP+10 µM EDTA) (data not shown). Under these conditions both native and recombinant MSP displayed prominent fungicidal activity.

The cytotoxicity of MSP was evaluated by the ability of 100 µM MSP_66–76_ or MSP_66–76E/Q_ to cause LDH release from HaCaT cells and lysis of erythrocytes. No significant LDH release or lysis was seen (Table S1), indicating that fungicidal MSP was not overly toxic to human cells.

**Figure 7:**
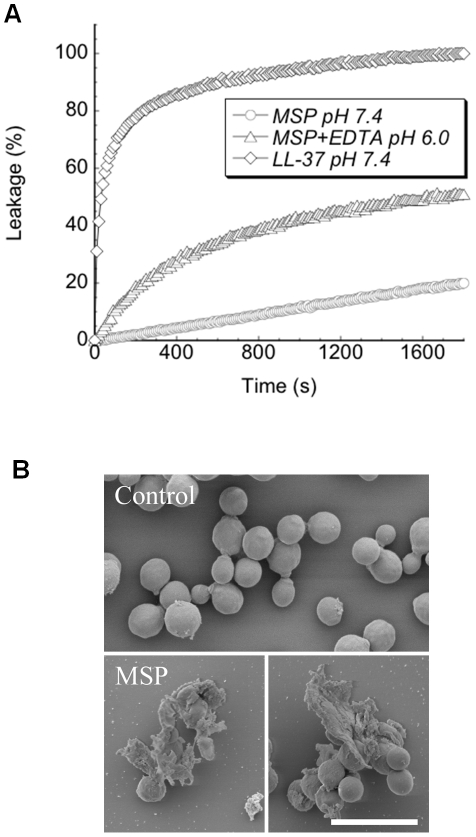
Liposome leakage experiments and electron microscopy. **A.** Liposome leakage experiment depicting the effects of 1 µM MSP or LL-37 on permeability of ergosterol-containing liposomes. Leakage is depicted as % of total leakage obtained by triton-X-100. **B.** Scanning electron microscopy of *C. albicans* before and after treatment with MSP. Scale bar represents 10 µm.

Since MSP did not display any pronounced antibacterial or cytotoxic activity, we investigated whether the selectivity towards fungi was due to a selective affinity towards membranes mimicking fungal membranes. While vesicle leakage induction by MSP was virtually absent for DOPC/cholesterol liposomes, which mimic mammalian cell membranes, membrane disruption was observed for DOPC/ergosterol liposomes, which mimic the fungal membrane (Table 2). Furthermore, increased membrane lysis was obtained at pH 6 (lowest possible assay pH) compared to 7.4 demonstrating that peptide/protein-induced liposome lysis is somewhat enhanced at the lower pH (Figure 7 A). EDTA caused a slight increase in the amplitude of membrane lysis by MSP and MSP_66–76_ at pH 6.0, while no effect was observed at pH 7.4. Control experiments with EDTA alone showed no liposome rupture. Taken together, the liposome results indicated that MSP had membrane-disruptive capacity, especially at acidic pH, but selectively for fungal-like membranes. Quantitatively, however, the effects were smaller than observed by potent antimicrobial peptides such as W-tagged peptides [13], LL-37 derivatives [14], or other potent AMPs [15].

**Table 2:** Liposome leakage activity of MSP and MSP_66–76_.

	DOPC/ergosterol	DOPC/ergosterol	DOPC/cholesterol
	pH 7.4	pH 6.0	pH 6.0
MSP	20±5	36±8	0
MSP+EDTA	20±6	53±5	4±2
MSP_66–76_	29±6	31±6	0
MSP_66–76_+EDTA	16±7	41±9	0

Liposome leakage is given as % ± standard deviation of the leakage obtained with Triton X-100 disruption of the liposomes.

To further substantiate membrane disruption being part of the fungicidal effect of MSP, scanning electron microscopy was performed. *C. albicans* incubated with buffer only were healthy looking and could be seen dividing, whereas *C. albicans* incubated with MSP and EDTA had aggregated with disrupted membranes causing cell content leakage (Figure 7 B). EDTA alone had no effect. Membrane disruption is, thus, a central part of the antifungal action of MSP.

## Discussion

While bacterial vaginosis is associated with frequent sexual intercourse, this is not the case with vaginal candidiasis [3]. Indeed penile-vaginal transmission of *Candida* is rare [2]. This difference between the sexual transmission of bacterial vaginosis and vaginal candidiasis is quite remarkable since seminal plasma contains potent antibacterial activity [4], [5], [6], [7]. Since *Candida* is controlled by innate immune factors [1], we investigated whether post coital seminal plasma contained innate immune factors with activity against *Candida*, explaining the low sexual transmission rate. We found that the acidic post coital seminal plasma had a not previously described potent candidacidal activity. Simple acidification endowed normal seminal plasma with similar potent candidacidal activity; while no significant antifungal activity was found in samples of vaginal fluid collected and diluted the same way as the post coital seminal plasma. The potent candidacidal activity was mediated by the prostate-derived protein, MSP. MSP is present in 0.5–1 mg/ml concentrations in seminal plasma [16], [17] and has no previously described antimicrobial function. The candidacidal activity of MSP and of seminal plasma was activated in the vagina by a novel calcium- and pH-dependent mechanism uniquely suited for the post coital vaginal milieu. When present in seminal plasma at neutral to slightly alkaline pH, the fungicidal activity of MSP was inhibited by calcium. When an ejaculate is deposited into the acidic vaginal milieu the pH remains neutral for a time, allowing the spermatozoa to reach the uterus without being damaged by the acidic vaginal pH [18]. However, after a few hours, vaginal pH drops back to acidic pH [19]. The level of free ionized calcium in seminal plasma is low compared to blood plasma, and, additionally, seminal plasma has a high calcium buffering capacity due to a high concentration of citrate [20]. The combination of low calcium levels and acidic pH unleash the candidacidal activity of MSP post coitally in the vagina. The fungicidal activity of both whole seminal plasma and purified MSP was unleashed by chelation of calcium by EDTA. This calcium inhibition at neutral pH was not relieved, even by extensive dialysis, revealing a strong association between MSP and calcium at neutral pH. Incubation at the low vaginal pH followed by dialysis endowed seminal plasma with fungicidal activity even at neutral pH. This indicated that at low calcium-levels and low pH, MSP no longer bound calcium and inhibition of the fungicidal activity was relieved. According to NMR-analysis calcium-binding to MSP was associated with glutamic acid residue E_71_. The theoretical pKa of glutamic acid is 4.1 and the electrostatic interaction between the positively charged calcium and the negatively charged glutamic acid residue will decrease as the pH is lowered towards the pKa of glutamic acid and could offer an explanation for the pH dependent activation of fungicidal activity. To the best of our knowledge, this is the first time that the activity of both an AMP and a seminal plasma protein has been described to be modulated in this calcium and pH-dependent manner.

The structure of the C-terminal part of MSP encompassing the fungicidal activity has no structure similarity to other known proteins [11]. AMPs are usually positively charged and hydrophobic and these characteristics are found in the fungicidal part of MSP (MSP_66–76_). Many AMPs with potent antifungal activity, like S100A8/S100A9, are also antibacterial [21]. However, we did not find any prominent activity against the bacterial species tested or any substantial cytotoxic activity against human cells. The liposome leakage experiments demonstrated that MSP selectively interacted with ergosterol-containing liposomes, mimicking fungal membranes, which offers an explanation of the selectivity of MSP against fungi and the lack of substantial cytotoxicity.

MSP has been demonstrated to decrease tumor growth in an *in vivo* model of prostate cancer [22]. This activity is localized mainly to MSP_31–45_ in the N-terminal domain of MSP, which has structural similarity to the fibronectin type 1 module [23]. By contrast, the effect on tumor growth is apparently unaffected by calcium, and moreover we determined that the fungicidal effect was localized to MSP_66–76_. Accordingly, the fungicidal effect is expected to be independent of the experimental effect on tumor growth.

Microbial surfaces are anionic with binding sites for divalent cations such as magnesium and calcium. The binding of these divalent cations stabilizes the microbial membranes. The positively charged AMPs displace the divalent cations resulting in membrane destabilization and uptake of the AMPs [24]. This process is termed self-promoted uptake and is important for the antimicrobial activity of AMPs. Consequently, a surplus of divalent cations like magnesium and calcium inhibits the antimicrobial activity of AMPs when included in antimicrobial assays. Importantly, the mechanism by which MSP was inhibited by calcium was entirely different. Firstly, the calcium-mediated inhibition of MSP was due to a direct interaction between the divalent cations and MSP and not with the microbial surface. Secondly, the effect is specific for calcium since magnesium in similar concentrations did not inhibit the fungicidal activity. This difference is underscored by the comparative experiments with the classical AMP, LL-37, where the antifungal activity did not display the pH-dependent inhibition by calcium as observed with MSP-derived peptides and whole seminal plasma.

Although innate immunity effector molecules like AMPs are found in various body fluids and tissues [25], elicitation of the innate immune responses, such as complement activation, is strictly controlled and only takes place locally [26]. The same is the case with proteolytic generation of AMPs where, for example, neutrophil-derived LL-37 is generated after exudation of the neutrophils into the tissues [27] and the semenogelin-derived peptides are generated after ejaculation [7]. Antimicrobial testing of mammalian AMPs is often performed at standard conditions around neutral pH. However, the environment of the mucosal surfaces in the human body varies greatly and so do the conditions where AMPs are active. Extremophiles produces AMPs with activity only at molar salt concentrations [28] – conditions rendering mammalian AMPs inactive. Appropriately, these AMPs are active in the environment of the extremophile. Similarly, MSP is a fungicidal agent uniquely adapted to the post coital vaginal milieu with the acidic pH and the low calcium concentration where MSP is deposited after sexual intercourse. The calcium- and pH-dependent activation of the antifungal activity of MSP is to the best of our knowledge the first description of a human AMP that demonstrates this strictly environmentally dependent behavior. It raises the question whether new antimicrobial properties of other known proteins could be identified if the specific environment of the proteins is taken into consideration when performing antimicrobial testing.

In conclusion MSP was identified as a novel candidacidal agent endowing seminal plasma with potent fungicidal activity after subjection to the acidic vaginal pH. The antifungal activity of MSP was unleashed in the vagina after sexual intercourse by a combination of the vaginal acidic pH and low calcium concentration. This represents a novel mechanism for regulation of antimicrobial activity of antimicrobial proteins uniquely suited for the post coital vaginal environment.

## Materials and Methods

### Ethic statement

All research was approved by the institutional review board at Lund University, University of Copenhagen, and University of Central Florida. Written informed consent was provided by the study participants.

### Reagents

Recombinant human β-microseminoprotein and polyclonal anti-β-microseminoprotein antibodies were from R&D Systems (Minneapolis, USA). HRP-conjugated antibodies were from DAKO (Glostrup, Denmark). Human β-microseminoprotein was purified as previously described [12]. Synthetic MSP-derived peptides were purchased from Shafer-N (Copenhagen, Denmark).

### Candidal strains and growth conditions

*Candida albicans strains:* ATCC 90028, CAF-1-2, SC5814, BM4f35II68, vaginal clinical isolates from Dept. of Clincal Microbiology, Skåne University Hospital, SO_303369 and SO_503471. *Candida parapsilosis strains:* ATCC 90018, BD1800, BD17837, and BM24/68/09. *Candida glabrata* strains: ATCC 90030, clinical isolates from Dept. of Clincal Microbiology, Skåne University Hospital, SO_3336, SO_3400 and SO_503413. All strains were inoculated from frozen culture and grown in Yeast extract-Peptone-Dextrose (YPD) (Sigma) and on Sabourand Dextrose Broth (SAB) (Difco) plates at 30°C with 5% (v/v) CO_2_. If not otherwise stated, ATCC 90028 was used for candidacidal experiments.

### Bacterial strains and growth conditions

*E. coli* (strain 37.4), *S. aureus* (strain 5120), *S. pyogenes* (M5 Manfredo), *S. agalactiae* (SB1), and *E. faecalis* (strain 2374) were grown in THY medium (Todd Hewitt with addition of yeast) and on THY agar plates at 37°C with 5% (v/v) CO_2_.

### Seminal plasma and vaginal fluid samples

Freshly ejaculated semen was collected from healthy volunteers and patients at Malmö University Hospital Fertility Clinic. The semen was allowed to liquefy for 1 h at room temperature followed by centrifugation at 1000× *g* at 4°C for 10 min. The supernatant was collected, aliquoted and stored at −20°C. Before the seminal plasma was used in a CFU assay it was centrifuged at 16000× *g* for 20 min at 4°C and dialyzed (MWCO 3500 Da) against 25 mM ammonium acetate pH 7 or pH 4 at 4°C.

To incubate seminal plasma at different pH a suitable amount of 1 M ammonium acetate pH 4, pH 5 or pH 6 was added and after incubation the sample was dialyzed as above.

Three samples of post coital seminal plasma from different donors were carefully aspirated by syringe from the vagina of healthy donors 8–10 hours following sexual intercourse and stored at −20°C as previously described [29]. Before use, the samples were centrifuged at 16000× *g* for 20 min at 4°C and the supernatants used for further experiments. The samples were dialyzed at neutral pH the same way as regular seminal plasma prior to use in CFU assays.

Two samples of semen instilled in the vagina of women undergoing insemination were carefully aspirated with syringe after 4 h [29]. These samples had a neutral pH.

For CFU assays, vaginal fluids were carefully aspirated with a syringe from the vagina of healthy women before insemination and stored at −20°C in the same manner as the post coital seminal plasma was collected. Before use it was centrifuged at 16000× *g* for 20 min at 4°C and the supernatant used for further experiments. Due to the small volumes, vaginal fluid was not dialyzed but buffer changed on a VivaSpin500 centrifugal device (3 kDa MWCO) (GE Healthcare) to 10 mM Tris-HCl, 5 mM glucose, pH 4.

### Gel overlay assay

Gel overlay assay was performed essentially as described [30]. Briefly, duplicate samples were run on native Acid Urea (AU-PAGE) gels in 5% (v/v) acetic acid at 100 V for 1 h 20 min. *Candida albicans* was grown over night, washed and resuspended in 10 mM NaPO_4_ pH 7.4 and approximately 1×10^5^ CFU were added to 12 ml melted underlay agarose (10 mM NaPO_4_, pH 7.4, 0.03% SAB, 1% agarose type 1 (Sigma, St. Louis, MO)) and poured into a square Petri dish. One AU-gel was stained with Coomassie brilliant blue and one AU gel was washed 3×4 min in 10 mM NaPO_4_, pH 7.4 and then placed on top of the agarose and incubated for 4 h at 37°C. The AU gel was then removed and an overlay agarose (6% SAB, 1% agarosetype 1 (Sigma)) was poured on top of the underlay and incubated over night at 30°C. To make the clearing zones more visible, the agarose was stained with Coomassie brilliant blue followed by destaining.

### Antimicrobial activity assay/Colony forming unit (CFU) assay

*Candida albicans* was grown over night, washed, and resuspended in 10 mM Tris, 5 mM glucose, pH 7.4 or pH 4. Approximately 4×10^3^ to 10^4^ CFU of *C. albicans* were incubated with the test substance in low or neutral pH buffer for 4 h at 37°C. After incubation, the suspension was diluted and plated. Colonies were counted after two days incubation of the plates at 30°C. Bacteria from overnight cultures were inoculated in fresh medium and grown for 3 h, washed in 10 mM Tris, 5 mM glucose pH 7.4. Approximately 8×10^4^ CFU were incubated with sample in 10 mM Tris, 5 mM glucose pH 7.4 or pH 4 for 1 h at 37°C, diluted, plated and incubated over night at 37°C and the colonies counted.

### Peptide identification

Seminal plasma samples were run on two identical AU-gels. One was stained with Coomassie Blue and the other was used in a gel overlay assay. Bands corresponding to the clearing zones in the agarose gel were cut out from the stained gel and sent to Alphalyse (Alphalyse A/S, Odense, Denmark) where the gel band was treated with trypsin and the resulting peptides analyzed by MALDI TOF/TOF mass spectrometry. The masses of the peptides were used to query sequence databases for proteins with matching peptide masses.

### NMR analysis

MSP_66–76_ was dissolved in milliQ-water and lyophilized twice. Two samples of 50 µM peptides were prepared in a volume of 600 µL each in 20 mM Tris-HCl, pH 7.4 with either 10 mM EDTA or 10 mM CaCl_2_. The samples were centrifuged for 5 min at 12000× *g* and transferred to 5 mm NMR tubes. 1D ^1^H NMR spectra were recorded on a Varian INOVA 750 (^1^H) MHz spectrometer with Z-field gradient with 1024 transients at 37°C. Spectra were transformed and analyzed using MesTREC and referenced to acetonitril (1.95 ppm).

### Scanning electron microscopy

*Candida* samples, approximately 2×10^4^ CFU per sample, were incubated for 5 h with buffer only or 20 µM MSP with 40 µM EDTA, applied to poly-L-lysine coated coverslips for 1 h, and subsequently fixed in 2.5% (v/v) glutaraldehyde in 0.15 M sodium cacodylate, pH 7.4 for 30 min at room temperature. Specimens were washed with cacodylate buffer, and dehydrated with an ascending ethanol series from 50% (v/v) to absolute ethanol. The specimens were then subjected to critical-point drying in carbon dioxide, with absolute ethanol as intermediate solvent, mounted on aluminum holders, sputtered with 30 nm palladium/gold and examined in a JEOL JSM-350 scanning electron microscope. A small volume from each of the samples was also plated to check antifungal activity.

### Liposome preparation and leakage experiments

Dry lipid films were prepared by dissolving dioleoylphosphatidylcholine (1,2-dioleoyl-sn-Glycero-3-phoshocholine, >99% purity, Avanti Polar Lipids, Alabaster, AL) (60 mol%) (DOPC) and either ergosterol or cholesterol (both >99% purity, Sigma) (40 mol%) in chlorofom, and then removing the solvent by evaporation under vacuum overnight. Subsequently, buffer (10 mM Tris, pH 7.4) was added together with 0.1 M carboxyfluorescein (CF) (Sigma, St Louis, MO). After hydration, the lipid mixture was subjected to eight freeze-thaw cycles consisting of freezing in liquid nitrogen and heating to 60°C. Unilamellar liposomes of about Ø140 nm were generated by multiple extrusions through polycarbonate filters (pore size 100 nm) mounted in a LipoFastminiextruder (Avestin, Ottawa, Canada) at 22°C. Untrapped CF was then removed by two gel filtrations (Sephadex G-50) at 22°C, with Tris buffer as eluent. CF release was determined by monitoring the emitted fluorescence at 520 nm from liposome dispersions (10 mM lipid in 10 mM Tris). An absolute leakage scale was obtained by disrupting the liposomes at the end of the experiment through addition of 0.8 mM Triton X100 (Sigma, St Louis, MO), causing 100% release and dequenching of CF. Although calcein is frequently used for pH-dependent leakage studies, the high charge of this dye has been noted to influence its leakage behavior in the presence of highly cationic peptides. Instead, therefore, CF was used as a leakage marker at both pH 6.0 and 7.4, avoiding pH-dependent fluorescence effects through neutralization prior to probing the limiting leakage in case of pH 6.0 leakage. Throughout, a SPEX-fluorolog 1650 0.22-m double spectrometer (SPEX Industries, Edison, NJ) was used for the liposome leakage assay. Measurements were performed in at least triplicate at 37°C.

### Statistical analysis

Statistical analysis was performed using SAS 9.2 software (SAS Institute, Cary, NC). Colony counts were log transformed. In order to choose the statistical method, interactions between the Treatment and Experiment were investigated. Treatments were screened by doing pair wise comparisons of each treatment to a control. Statistical tests used for the comparisons included MIXED procedure and LSMEANS statement. All tests where *P-value*<.05 were considered to indicate statistical significance. P-values were not adjusted for the multiple comparisons.

## Supporting Information

Dataset S1 **Statistical analysis of CFU assays.**
(DOC)

Figure S1 **Activity of dialyzed seminal plasma against** ***Candida*** **strains.** The candidacidal activity of 1/50 dilution of dialyzed seminal plasma (2%) was tested against strains of *C. albicans*, *C. parapsilosis*, and C. *glabrata*. At pH 4, more than 10 colonies were found in 1000-fold dilution of the control samples, but no colonies were found in 10-fold dilution of the samples of *C. albicans* and C. *parapsilosis* treated with dialyzed seminal plasma corresponding to a more than 3 log reduction. In contrast, no significant killing was observed at pH 7 or in any of the *C. glabrata* samples. All results are shown as the average from three independent experiments. ** denotes p<0.001. Error bars indicate standard deviations. Further details regarding the statistical analysis are found in Dataset S1. (Black bars: dialyzed seminal plasma, white bars: control).
(TIF)

Figure S2 **Candidacidal activities of MSP_66–76E/Q_ and MSP_66–76D/A_.** **A.** While the antifungal activity of MSP_66–76E/Q_ was not inhibited by calcium (0.5 mM) at acidic pH, the activity was still inhibited at 50–100 µM calcium at neutral pH. However, even at neutral pH the activity was more calcium resistant than MSP_66–76_ since this peptide only was active at neutral pH with equimolar concentration of EDTA. Similar inhibition by calcium at neutral pH was found for MSP_66–76D/A_ where the aspartic acid (D) adjacent to the glutamic acid was substituted with an alanine (A). **B.** The antifungal activity of MSP_66–76D/A_ was not inhibited by even 0.5 mM calcium at acidic pH. Coordination of calcium in proteins typically involves six different atoms normally originating from up to six different residues and including backbone carbonyls. It is therefore not surprising that single or even double mutation in the peptide preserved calcium binding, but with an expected lower affinity.
(TIF)

Figure S3 **Seize exclusion chromatography of MSP.** Native MSP was analyzed by seize exclusion chromatography on a HiPrep 16/60 Sephacryl S 100 column in the presence of 1 mM calcium or 1 mM EDTA in Trisbuffer (50 mM Tris, 150 mM NaCl, pH 7.5).
(TIF)

Figure S4 **Antifungal activity of peptide derived from porcine MSP.** Porcine MSP was tested in CFU assay at pH 4 with and without EDTA. All results are shown as the average from three independent experiments. ** denotes p<0.001. Error bars indicate standard deviations. Further details regarding the statistical analysis are found in Dataset S1.
(TIF)

Table S1 **Cytoxic and hemolytic Effect of MSP_66–76E/Q_ and MSP_66–76_.** HaCaT cells were treated with 100 µM of MSP_66–76E/Q_ and MSP_66–76_ over night. The release of LDH was subsequently determined by colorimetric assay and depicted as percent of LDH release of cells treated with 2% Triton- X100. Erythrocytes were treated for 1 ½ hour with 100 µM of MSP_66–76E/Q_ and MSP_66–76_ followed by centrifugation. Lysis was determined by measuring hemoglobin in the supernatant by measuring the absorbance at 550 nm. Results are depicted as % of lysis compared to lysis obtained by 2% Triton-X100.
(TIF)
