# Supplementary material for: β-Microseminoprotein Endows Post Coital Seminal Plasma with Potent Candidacidal Activity by a Calcium- and pH-Dependent Mechanism
Source: PLoS Pathog. 2012 Apr 5;8(4):e1002625. doi: 10.1371/journal.ppat.1002625 (PMC3320615; doi:10.1371/journal.ppat.1002625)
Supplement: Dataset S1 — Statistical analysis of CFU assays. (DOC) [file ppat.1002625.s001.doc]

**Dataset S1 - statistical analysis**

In the large number of the figures, the interaction between the treatment and the experiment was significant, suggesting that the magnitude of differences between the treatment means (or between the specific treatment and the control mean) depends on which experiment they have been observed from (data not shown). Hence, testing for the overall differences between the treatment and the control averaged over all the experiments is not appropriate. Instead, log of each treatment mean is compared to the log of mean of control for each experiment.

***“Estimate” is the estimate of the difference between means of log values of treatment 1 and treatment 2***

***Figure 1 A***

| **Experiment** | **Treatment 1** | **Treatment 2** | **Estimate** | **P-value** |
| --- | --- | --- | --- | --- |
| 1 | 1% PC SP | Control | -2.8601 | <.0001 |
| 2 | 1% PC SP | Control | -3.6322 | <.0001 |
| 3 | 1% PC SP | Control | -3.1671 | <.0001 |

**Killing of *C. albicans* with 1% PC DSP a pH 7.4.**

Three independent experiments were performed. Significant difference of mean colony counts between control and 1 % PC was found in all experiments (p<0.001).

| **Experiment** | **Treatment 1** | **Treatment 2** | **Estimate** | **P-value** |
| --- | --- | --- | --- | --- |
| 1 | 5% PC SP | Control | -4.0280 | <.0001 |
| 2 | 5% PC SP | Control | -4.0016 | <.0001 |
| 3 | 5% PC SP | Control | -4.1417 | <.0001 |

**Killing of *C. albicans* with 5% PC DSP at pH 7.4.**

Three independent experiments were performed. Significant difference of mean colony counts between control and 5 % PC SP was found in all experiments (p<0.001).

| **Experiment** | **Treatment1** | **Treatment2** | **Estimate** | **P-value** |
| --- | --- | --- | --- | --- |
| 1 | 1% PC SP | Control | -4.2788 | <.0001 |
| 2 | 1% PC SP | Control | -4.0276 | <.0001 |
| 3 | 1% PC SP | Control | -3.9405 | <.0001 |

**Killing of *C. albicans* with 1% PC DSP at pH 4.**

Three independent experiments were performed. Significant difference of mean colony counts between control and 1 % PC SP was found in all experiments (p<0.001).

| **Experiment** | **Treatment1** | **Treatment2** | **Estimate** | **P-value** |
| --- | --- | --- | --- | --- |
| 1 | 5% PC SP | Control | -4.4257 | <.0001 |
| 2 | 5% PC SP | Control | -5.2879 | <.0001 |
| 3 | 5% PC SP | Control | -5.2881 | <.0001 |

**Killing of *C. albicans* with 5% PC DSP at pH 4.**

Three independent experiments were performed. Significant difference of mean colony counts between control and 1 % PC SP was found in all experiments (p<0.001).

***Figure 1B***

| **Experiment** | **Treatment1** | **Treatment2** | **Estimate** | **P-value** |
| --- | --- | --- | --- | --- |
| 1 | 2 % VF | control | 0.1708 | 0.1500 |
| 2 | 2 % VF | control | -1.0665 | <.0001 |
| 3 | 2 % VF | control | -0.00338 | 0.9766 |
| 1 | 5% VF | control | -0.5977 | <.0001 |
| 2 | 5% VF | control | -0.9206 | <.0001 |
| 3 | 5% VF | control | 0.1180 | 0.3123 |

**Killing of *C. albicans* with 2% VF and 5% VF at pH 4.**

Three independent experiments were performed. No significant difference of mean colony counts between control and VF was found.

| **Experiment** | **Treatment 1** | **Treatment2** | **Estimate** | **P-value** |
| --- | --- | --- | --- | --- |
| 1 | 2 % DVF | control | 0.2320 | 0.2806 |
| 2 | 2 % DVF | control | -0.04218 | 0.8420 |
| 3 | 2 % DVF | control | -0.1536 | 0.4709 |
| 1 | 5% DVF | control | 0.04740 | 0.8227 |
| 2 | 5% DVF | control | 0.1556 | 0.4651 |
| 3 | 5% DVF | control | 0.2648 | 0.2204 |

**Killing of *C. albicans* with 2% DVF and 5% DVF at pH 4.**

Three independent experiments were performed. No significant difference of mean colony counts between control and DVF was found.***Figure 1C***

| **Experiment** | **Treatment1** | **Treatment2** | **Estimate** | **P-value** |
| --- | --- | --- | --- | --- |
| 1 | 1% DSP | Control | -0.2062 | 0.3730 |
| 2 | 1% DSP | Control | -0.2453 | 0.2910 |
| 3 | 1% DSP | Control | -0.1934 | 0.4031 |
| 1 | 1% SP | Control | 1.0544 | 0.0001 |
| 2 | 1% SP | Control | 1.2421 | <.0001 |
| 3 | 1% SP | Control | 1.3450 | <.0001 |
| 1 | 1% pH 4 inc DSP | Control | -2.8558 | <.0001 |
| 2 | 1% pH 4 inc DSP | Control | -3.9984 | <.0001 |
| 3 | 1% pH 4 inc DSP | Control | -3.6821 | <.0001 |

**Killing of *C. albicans* with 1% SP, DSP, and pH 4 inc DSP at pH 7.4.**

Three independent experiments were performed. Significant difference of mean colony counts between control and 1 % pH 4 inc SP was found in all experiments (p<0.001) but no difference between mean colony counts between control and 1% SP and 1% DSP. SP actually significantly increased the colony counts compared to controls.

| **Experiment** | **Treatment1** | **Treatment2** | **Estimate** | **P-value** |
| --- | --- | --- | --- | --- |
| 1 | 5% DSP | Control | 1.1867 | <.0001 |
| 2 | 5% DSP | Control | 1.6999 | <.0001 |
| 3 | 5% DSP | Control | 0.9042 | <.0001 |
| 1 | 5% SP | Control | 1.3820 | <.0001 |
| 2 | 5% SP | Control | 1.7576 | <.0001 |
| 3 | 5% SP | Control | 1.1479 | <.0001 |

**Killing of *C. albicans* with 5% SP and DSP at pH 7.4.**

Three independent experiments were performed. Significant difference of mean colony counts between control and 5% DSP and 5% SP was found in all experiments (p<0.001) demonstrating that treatment with 5% SP and 5% DSP **increased** the colony compared to controls.

| **Experiment** | **Treatment1** | **Treatment2** | **Estimate** | **P-value** |
| --- | --- | --- | --- | --- |
| 1 | 5% pH 4 inc DSP | Control | -4.0280 | <.0001 |
| 2 | 5% pH 4 inc DSP | Control | -4.0016 | <.0001 |
| 3 | 5% pH 4 inc DSP | Control | -4.1417 | <.0001 |

**Killing of *C. albicans* with 5% ph 4 inc DSP at pH 7.4.**

Three independent experiments were performed. Significant difference of mean colony counts between control and 5 % pH 4 inc SP was found in all experiments (p<0.001).

| **Experiment** | **Treatment1** | **Treatment2** | **Estimate** | **P-value** |
| --- | --- | --- | --- | --- |
| 1 | 1% SP | Control | -4.5842 | <.0001 |
| 2 | 1% SP | Control | -3.8359 | <.0001 |
| 3 | 1% SP | Control | -2.6203 | <.0001 |
| 1 | 1% pH 4 inc DSP | Control | -4.8153 | <.0001 |
| 2 | 1% pH 4 inc DSP | Control | -3.4696 | <.0001 |
| 3 | 1% pH 4 inc DSP | Control | -4.2675 | <.0001 |

**Killing of *C. albicans* with 1% SP and 1% pH 4 inc DSP at pH 4.**

Three independent experiments were performed. Significant difference of mean colony counts between control and 1 % pH 4 inc SP and 1 % SP was found in all experiments (p<0.001).

| **Experiment** | **Treatment1** | **Treatment2** | **Estimate** | **P-value** |
| --- | --- | --- | --- | --- |
| 1 | 1% DSP | Control | -6.0575 | <.0001 |
| 2 | 1% DSP | Control | -4.5811 | <.0001 |
| 3 | 1% DSP | Control | -4.2975 | <.0001 |

**Killing of *C. albicans* with 1% DSP at pH 4.**

Three independent experiments were performed. Significant difference of mean colony counts between control and 1 % DSP was found in all experiments (p<0.001).

| **Experiment** | **Treatment1** | **Treatment2** | **Estimate** | **P-value** |
| --- | --- | --- | --- | --- |
| 1 | 5% DSP | Control | -4.4257 | <.0001 |
| 2 | 5% DSP | Control | -5.2879 | <.0001 |
| 3 | 5% DSP | Control | -5.2881 | <.0001 |
| 1 | 5% SP | Control | -0.5077 | <.0001 |
| 2 | 5% SP | Control | -1.0176 | <.0001 |
| 3 | 5% SP | Control | -1.2086 | <.0001 |
| 1 | 5% pH 4 inc DSP | Control | -4.4257 | <.0001 |
| 2 | 5% pH 4 inc DSP | Control | -5.2879 | <.0001 |
| 3 | 5% pH 4 inc DSP | Control | -5.2881 | <.0001 |

**Killing of *C. albicans* with 5% SP, 5% DSP and 5% pH 4 inc DSP at pH 4.**

Three independent experiments were performed. Significant difference of mean colony counts between control and 4 % pH 4 inc SP , 5 % DSP and 5 % SP was found in all experiments (p<0.001).

***Figure 2C***

| **Experiment** | **Treatment1** | **Treatment2** | **Estimate** | **P-value** |
| --- | --- | --- | --- | --- |
| 1 | Cont | vas | 4.2461 | <.0001 |
| 2 | Cont | vas | 4.0273 | <.0001 |
| 3 | Cont | vas | 4.3423 | <.0001 |

**Killing of *C. albicans* with DSP from vasectomised patient at pH 4.**

Three independent experiments were performed. Significant difference of mean colony counts between control and seminal plasma from vasectomised patient (vas) was found in all experiments (p<0.001).

| **Experiment** | **Treatment1** | **Treatment2** | **Estimate** | **P-value** |
| --- | --- | --- | --- | --- |
| 1 | Cont | Ves def | 4.0829 | <.0001 |
| 2 | Cont | Ves def | 4.1408 | <.0001 |
| 3 | Cont | Ves def | 4.4843 | <.0001 |

**Killing of *C. albicans* with DSP from patient with deficient seminal vesicles at pH 4.**

Three independent experiments were performed. Significant difference of mean colony counts between control and seminal plasma from patient with deficient seminal vesicles (Ves def) was found in all experiments (p<0.001).

***Figure 3A***

| **Experiment** | **Treatment1** | **Treatment2** | **Estimate** | **P-value** |
| --- | --- | --- | --- | --- |
| 1 | Control | EDTA | 0.3309 | 0.0016 |
| 1 | Control | MSP | -0.06377 | 0.4826 |
| 2 | Control | EDTA | -0.2077 | 0.0313 |
| 2 | Control | MSP | -0.4481 | <.0001 |
| 3 | Control | EDTA | -0.01878 | 0.8352 |
| 3 | Control | MSP | -0.4892 | <.0001 |

**Killing of *C. albicans* with MSP and EDTA at pH 4.**

Overall no significant difference of mean colony counts between control and MSP or EDTA was found.

| **Obs** | **Experiment** | **Treatment1** | **Treatment2** | **Estimate** | **P-value** |
| --- | --- | --- | --- | --- | --- |
| **1** | 1 | Control | MSP + EDTA | 4.1638 | <.0001 |
| **2** | 1 | Control | MSP+EDTA/Ca | -0.9947 | 0.0018 |
| **3** | 1 | Control | MSP+EDTA/Mg | 1.9399 | <.0001 |
| **4** | 2 | Control | MSP + EDTA | 4.7089 | <.0001 |
| **5** | 2 | Control | MSP+EDTA/Ca | -0.9876 | 0.0019 |
| **6** | 2 | Control | MSP+EDTA/Mg | 2.1624 | <.0001 |
| **7** | 3 | Control | MSP + EDTA | 4.1911 | <.0001 |
| **8** | 3 | Control | MSP+EDTA/Ca | -1.0805 | 0.0008 |
| **9** | 3 | Control | MSP+EDTA/Mg | 1.6349 | <.0001 |

**Killing of *C. albicans* with MSP+EDTA, MSP+EDTA/Ca, and MSP+EDTA/Mg at pH 4.**

Three independent experiments were performed. Significant difference (reduction) of mean colony counts was found between control and MSP+EDTA and MSP+EDTA/Mg (p< 0.001). The significant difference in mean colony counts between control and MSP+EDTA/Ca were due to increased mean colony counts in samples with MSP+EDTA/Ca.

***Figure 3B***

| **Experiment** | **Treatment1** | **Treatment2** | **Estimate** | **P-value** |
| --- | --- | --- | --- | --- |
| 1 | Cont | DSP | 5.1947 | <.0001 |
| 2 | Cont | DSP | 2.3791 | <.0001 |
| 3 | Cont | DSP | 5.7732 | <.0001 |
| 1 | Cont | DSP + Ca | -0.3302 | 0.2407 |
| 2 | Cont | DSP + Ca | -0.3941 | 0.1639 |
| 3 | Cont | DSP + Ca | -0.5665 | 0.0500 |
| 1 | Cont | DSP + Mg | 3.9488 | <.0001 |
| 2 | Cont | DSP + Mg | 3.4898 | <.0001 |

**Killing of *C. albicans* with Dia SP, Dia SP + Mg, and Dia SP + Ca at pH 4.**

Three independent experiments were performed. Significant difference (reduction) of mean colony counts was found between control and DSP and DSP + Mg (p< 0.001). In contrast there was no significant difference in mean colony counts between control and DSP + Ca.

***Figure 3 C***

| **Experiment** | **Treatment** | **_Treatment** | **Estimate** | **P-value** |
| --- | --- | --- | --- | --- |
| 1 | Control | EDTA DSP | 2.1292 | <.0001 |
| 2 | Control | EDTA DSP | 2.4596 | <.0001 |
| 3 | Control | EDTA DSP | 1.2401 | <.0001 |

**Killing of *C. albicans* with EDTA-treated DSP at pH 7.4.**

Three independent experiments were performed. Significant difference (reduction) of mean colony counts was found between control and EDTA-treated DSP (p< 0.001).

| **Experiment** | **Treatment1** | **Treatment2** | **Estimate** | **P-value** |
| --- | --- | --- | --- | --- |
| 1 | Control | EDTA DSP | 4.0388 | <.0001 |
| 2 | Control | EDTA DSP | 4.2322 | <.0001 |
| 3 | Control | EDTA DSP | 5.3386 | <.0001 |

**Killing of *C. albicans* with EDTA-treated DSP at pH 4.**

Three independent experiments were performed. Significant difference (reduction) of mean colony counts was found between control and EDTA-treated DSP (p< 0.001).

***Figure 4A***

| **Experiment** | **Treatment1** | **Treatment2** | **Estimate** | **P-value** |
| --- | --- | --- | --- | --- |
| 1 | Control | MSP1 | -0.6949 | 0.0002 |
| 2 | Control | MSP1 | -0.6938 | 0.0002 |
| 3 | Control | MSP1 | -0.2057 | 0.2454 |
| 1 | Control | MSP1+EDTA | -0.3564 | 0.0458 |
| 2 | Control | MSP1+EDTA | -0.5558 | 0.0022 |
| 3 | Control | MSP1+EDTA | -0.1369 | 0.4384 |
| 1 | Control | MSP2 | -0.4638 | 0.0099 |
| 2 | Control | MSP2 | -0.7678 | <.0001 |
| 3 | Control | MSP2 | 0.2466 | 0.1644 |
| 1 | Control | MSP2+EDTA | 0.5727 | 0.0016 |
| 2 | Control | MSP2+EDTA | 0.2951 | 0.0970 |
| 3 | Control | MSP2+EDTA | 0.5877 | 0.0012 |
| 1 | Control | MSP3 | -0.2487 | 0.1609 |
| 2 | Control | MSP3 | -0.7316 | <.0001 |
| 3 | Control | MSP3 | -0.09956 | 0.5729 |
| 1 | Control | MSP3+EDTA | 0.09392 | 0.5948 |
| 2 | Control | MSP3+EDTA | 0.05166 | 0.7697 |
| 3 | Control | MSP3+EDTA | -0.02824 | 0.8728 |
| 1 | Control | MSP4 | -0.5304 | 0.0033 |
| 2 | Control | MSP4 | -0.8100 | <.0001 |
| 3 | Control | MSP4 | -0.2155 | 0.2239 |
| 1 | Control | MSP4+EDTA | -0.3731 | 0.0367 |
| 2 | Control | MSP4+EDTA | -0.4012 | 0.0250 |
| 3 | Control | MSP4+EDTA | -0.1411 | 0.4246 |
| 1 | Control | MSP5 | 0.5704 | 0.0017 |
| 2 | Control | MSP5 | 0.3334 | 0.0613 |
| 3 | Control | MSP5 | 0.6913 | 0.0002 |
| 1 | Control | MSP5+EDTA | 2.0492 | <.0001 |
| 2 | Control | MSP5+EDTA | 4.5365 | <.0001 |
| 3 | Control | MSP5+EDTA | 3.0952 | <.0001 |
| 1 | Control | MSP6 | -0.00322 | 0.9854 |
| 2 | Control | MSP6 | 0.07848 | 0.6566 |
| 3 | Control | MSP6 | 0.9139 | <.0001 |
| 1 | Control | MSP6+EDTA | 1.6087 | <.0001 |
| 2 | Control | MSP6+EDTA | 3.2325 | <.0001 |
| 3 | Control | MSP6+EDTA | 0.6931 | 0.0002 |

**Killing of *C. albicans* with overlapping MSP peptides at pH 4.**

Three independent experiments were performed. Significant difference (reduction) of mean colony counts was found between control and MSP5 + EDTA and MSP6 + EDTA (p< 0.001). No significant reduction in mean colony count was found with treatment with the other peptides compared to control.

***Figure 4 D***

| **Experiment** | **Treatment1** | **Treatment2** | **Estimate** | **P-value** |
| --- | --- | --- | --- | --- |
| 1 | Control | MSP_66-76_ | 5.5432 | <.0001 |
| 2 | Control | MSP_66-76_ | 4.1263 | <.0001 |
| 3 | Control | MSP_66-76_ | 2.8985 | <.0001 |
| 4 | Control | MSP_66-76_ | 4.7326 | <.0001 |
| 1 | Control | MSP_66-76_+Ca | 1.0371 | 0.0006 |
| 2 | Control | MSP_66-76_+Ca | 0.7970 | 0.0059 |
| 3 | Control | MSP_66-76_+Ca | -0.5619 | 0.0458 |
| 4 | Control | MSP_66-76_+Ca | 0.4135 | 0.1360 |
| 1 | Control | MSP_66-76_+Mg | 5.4081 | <.0001 |
| 2 | Control | MSP_66-76_+Mg | 4.1263 | <.0001 |
| 3 | Control | MSP_66-76_+Mg | 2.8985 | <.0001 |
| 4 | Control | MSP_66-76_+Mg | 5.0596 | <.0001 |

**Killing of *C. albicans* with MSP_66-76_, MSP_66-76_+ Mg, and Dia MSP_66-76_ + Ca at pH 4.**

Three independent experiments were performed. Significant difference (reduction) of mean colony counts was found between control and MSP_66-76_ and MSP_66-76_ + Mg (p< 0.001). No significant reduction in mean colony count was found with treatment with MSP_66-76_ + Ca.

***Figure*** 5A

| **Experiment** | **Treatment 1** | **Treatment2** | **Estimate** | **P-value** |
| --- | --- | --- | --- | --- |
| 1 | Control | MSP_66-76_ | 5.2769 | <.0001 |
| 2 | Control | MSP_66-76_ | 4.2266 | <.0001 |
| 3 | Control | MSP_66-76_ | 2.8351 | <.0001 |
| 4 | Control | MSP_66-76_ | 4.2317 | <.0001 |
| 5 | Control | MSP_66-76_ | 5.0824 | <.0001 |
| 1 | Control | MSP_66-76_+ 100 Ca | 0.07455 | 0.8020 |
| 2 | Control | MSP_66-76_+ 100 Ca | -0.1459 | 0.5836 |
| 3 | Control | MSP_66-76_+ 100 Ca | -0.4793 | 0.0754 |
| 4 | Control | MSP_66-76_+ 100 Ca | -0.1979 | 0.4577 |
| 5 | Control | MSP_66-76_+ 100 Ca | -0.3209 | 0.2304 |
| 1 | Control | MSP_66-76_+ 200 Ca | 0.4296 | 0.1520 |
| 2 | Control | MSP_66-76_+ 200 Ca | -0.6341 | 0.0198 |
| 3 | Control | MSP_66-76_+ 200 Ca | -0.7050 | 0.0100 |
| 4 | Control | MSP_66-76_+ 200 Ca | -0.2560 | 0.3376 |
| 5 | Control | MSP_66-76_+ 200 Ca | 0.04894 | 0.8540 |
| 1 | Control | MSP_66-76_+ 50 Ca | 0.4123 | 0.1689 |
| 2 | Control | MSP_66-76_+ 50 Ca | -0.3545 | 0.1858 |
| 3 | Control | MSP_66-76_+ 50 Ca | -0.7827 | 0.0045 |
| 4 | Control | MSP_66-76_+ 50 Ca | 0.2633 | 0.3242 |
| 5 | Control | MSP_66-76_+ 50 Ca | 0.02251 | 0.9325 |
| 1 | Control | MSP_66-76_+ 75 Ca | 0.2228 | 0.4547 |
| 2 | Control | MSP_66-76_+ 75 Ca | -0.4921 | 0.0681 |
| 3 | Control | MSP_66-76_+ 75 Ca | -0.1876 | 0.4815 |
| 4 | Control | MSP_66-76_ + 75 Ca | -0.2469 | 0.3549 |
| 5 | Control | MSP_66-76_+ 75 Ca | -0.2312 | 0.3861 |

**Killing of C. albicans with MSP_66-76_ at pH 7.4 with increasing calcium concentrations.**

Five independent experiments were performed. Significant difference (reduction) of mean colony counts was found between control and MSP_66-76_ with no calcium (p< 0.001). No significant reduction in mean colony count was found with treatment with calcium treatment.

| **Experiment** | **Treatment 1** | **Treatment2** | **Estimate** | **P-value** |
| --- | --- | --- | --- | --- |
| 1 | Control | MSP_66-76_ | 5.4425 | <.0001 |
| 2 | Control | MSP_66-76_ | 4.7863 | <.0001 |
| 3 | Control | MSP_66-76_ | 3.5454 | <.0001 |
| 4 | Control | MSP_66-76_ | 4.0909 | <.0001 |
| 5 | Control | MSP_66-76_ | 3.8004 | <.0001 |
| 1 | Control | MSP_66-76_+ 100 Ca | 0.6973 | 0.0711 |
| 2 | Control | MSP_66-76_+ 100 Ca | 1.1772 | 0.0029 |
| 3 | Control | MSP_66-76_+ 100 Ca | 0.01412 | 0.9704 |
| 4 | Control | MSP_66-76_+ 100 Ca | 1.0688 | 0.0066 |
| 5 | Control | MSP_66-76_+ 100 Ca | 0.1005 | 0.7920 |
| 1 | Control | MSP_66-76_+ 200 Ca | 1.0661 | 0.0067 |
| 2 | Control | MSP_66-76_+ 200 Ca | 0.6082 | 0.1143 |
| 3 | Control | MSP_66-76_+ 200 Ca | -0.8572 | 0.0276 |
| 4 | Control | MSP_66-76_+ 200 Ca | 0.6184 | 0.1085 |
| 5 | Control | MSP_66-76_+ 200 Ca | -1.1924 | 0.0026 |
| 1 | Control | MSP_66-76_+ 50 Ca | 2.5872 | <.0001 |
| 2 | Control | MSP_66-76_+ 50 Ca | 2.7886 | <.0001 |
| 3 | Control | MSP_66-76_+ 50 Ca | 1.3256 | 0.0009 |
| 4 | Control | MSP_66-76_+ 50 Ca | 2.0744 | <.0001 |
| 5 | Control | MSP_66-76_+ 50 Ca | 1.7552 | <.0001 |
| 1 | Control | MSP_66-76_+ 75 Ca | 2.5170 | <.0001 |
| 2 | Control | MSP_66-76_+ 75 Ca | 1.9220 | <.0001 |
| 3 | Control | MSP_66-76_+ 75 Ca | -0.3426 | 0.3704 |
| 4 | Control | MSP_66-76_ + 75 Ca | 1.5746 | 0.0001 |
| 5 | Control | MSP_66-76_+ 75 Ca | 1.0684 | 0.0066 |

**Killing of C. albicans wih MSP_66-76_ at pH 4 with increasing calcium concentrations.**

Five independent experiments were performed. Significant difference (reduction) of mean colony counts was found between control and MSP_66-76_ and MSP_66-76_ with 50 μM (p<0.001). Significant difference (reduction) of mean colony counts was found between control and MSP_66-76_ with 75 μM in four out of five experiments.

In all other conditions there were no significant difference between mean colony counts of control and samples with MSP_66-76_ and calcium

***Figure 5B***

| **Obs** | **Experiment** | **Treatment** | **_Treatment** | **Estimate** | **Probt** |
| --- | --- | --- | --- | --- | --- |
| **1** | 1 | Control | EDTA DSP | 2.1292 | <.0001 |
| **2** | 2 | Control | EDTA DSP | 2.4596 | <.0001 |
| **3** | 3 | Control | EDTA DSP | 1.2401 | <.0001 |
| **4** | 1 | Control | EDTA DSP + 100 Ca | -2.0591 | <.0001 |
| **5** | 2 | Control | EDTA DSP + 100 Ca | -1.6602 | <.0001 |
| **6** | 3 | Control | EDTA DSP + 100 Ca | -2.2662 | <.0001 |
| **7** | 1 | Control | EDTA DSP + 200 Ca | -2.1993 | <.0001 |
| **8** | 2 | Control | EDTA DSP + 200 Ca | -1.7539 | <.0001 |
| **9** | 3 | Control | EDTA DSP + 200 Ca | -2.0147 | <.0001 |
| **10** | 1 | Control | EDTA DSP + 25 Ca | 1.8022 | <.0001 |
| **11** | 2 | Control | EDTA DSP + 25 Ca | 2.0742 | <.0001 |
| **12** | 3 | Control | EDTA DSP + 25 Ca | 1.4266 | <.0001 |
| **13** | 1 | Control | EDTA DSP + 50 Ca | -0.2689 | 0.1144 |
| **14** | 2 | Control | EDTA DSP + 50 Ca | 1.9998 | <.0001 |
| **15** | 3 | Control | EDTA DSP + 50 Ca | -1.8230 | <.0001 |
| **16** | 1 | Control | EDTA DSP + 75 Ca | -2.2494 | <.0001 |
| **17** | 2 | Control | EDTA DSP + 75 Ca | -1.2297 | <.0001 |
| **18** | 3 | Control | EDTA DSP + 75 Ca | -2.3441 | <.0001 |

**Killing of *C. albicans* with EDTA DSP at pH 7.4 with increasing calcium concentrations.**

Three independent experiments were performed. Significant difference (reduction) of mean colony counts was found between control and EDTA DSP and EDTA DSP 25 μM calcium (p<0.001). In all other conditions there were no significant **reduction** in mean colony count between mean colony of control and samples with EDTA DSP and calcium.

| **Experiment** | **Treatment1** | **Treatment2** | **Estimate** | **P-value** |
| --- | --- | --- | --- | --- |
| 1 | Control | EDTA DSP | 4.0388 | <.0001 |
| 2 | Control | EDTA DSP | 4.2322 | <.0001 |
| 3 | Control | EDTA DSP | 5.3386 | <.0001 |
| 1 | Control | EDTA DSP + 100 Ca | 4.8671 | <.0001 |
| 2 | Control | EDTA DSP + 100 Ca | 4.4633 | <.0001 |
| 3 | Control | EDTA DSP + 100 Ca | 4.8765 | <.0001 |
| 1 | Control | EDTA DSP + 200 Ca | 1.6153 | <.0001 |
| 2 | Control | EDTA DSP + 200 Ca | 2.7800 | <.0001 |
| 3 | Control | EDTA DSP + 200 Ca | 3.0497 | <.0001 |
| 1 | Control | EDTA DSP + 25 Ca | 4.8671 | <.0001 |
| 2 | Control | EDTA DSP + 25 Ca | 4.3281 | <.0001 |
| 3 | Control | EDTA DSP + 25 Ca | 5.1076 | <.0001 |
| 1 | Control | EDTA DSP + 50 Ca | 4.1347 | <.0001 |
| 2 | Control | EDTA DSP + 50 Ca | 3.9268 | <.0001 |
| 3 | Control | EDTA DSP + 50 Ca | 5.1076 | <.0001 |
| 1 | Control | EDTA DSP + 75 Ca | 4.1739 | <.0001 |
| 2 | Control | EDTA DSP + 75 Ca | 4.6943 | <.0001 |
| 3 | Control | EDTA DSP + 75 Ca | 5.1076 | <.0001 |

**Killing of C. albicans EDTA DSP at pH 4 with increasing calcium concentrations.**

Three independent experiments were performed. Significant difference (reduction) of mean colony counts was found between control and all conditions with EDTA DSP with and without calcium (p<0.001).

***Figure 5C***

| **Experiment** | **Treatment1** | **Treatment2** | **Estimate** | **P-value** |
| --- | --- | --- | --- | --- |
| 1 | Control | PC DSP | 4.5648 | <.0001 |
| 1a | Control | PC DSP | 2.8239 | <.0001 |
| 2 | Control | PC DSP | 3.9717 | <.0001 |
| 2a | Control | PC DSP | 2.1251 | <.0001 |
| 3 | Control | PC DSP | 4.6203 | <.0001 |
| 3a | Control | PC DSP | 3.2767 | <.0001 |
| 1a | Control | PC DSP + 100 Ca | -0.4986 | 0.0513 |
| 2a | Control | PC DSP + 100 Ca | -0.9938 | 0.0008 |
| 3a | Control | PC DSP + 100 Ca | -0.9603 | 0.0004 |
| 1a | Control | PC DSP + 200 Ca | -0.9456 | 0.0004 |
| 2a | Control | PC DSP + 200 Ca | -1.4288 | <.0001 |
| 3a | Control | PC DSP + 200 Ca | -1.2977 | <.0001 |
| 1 | Control | PC DSP + 25 Ca | 1.2543 | <.0001 |
| 2 | Control | PC DSP + 25 Ca | 1.4894 | <.0001 |
| 3 | Control | PC DSP + 25 Ca | 1.1609 | <.0001 |
| 1 | Control | PC DSP + 50 Ca | 0.4002 | 0.1152 |
| 2 | Control | PC DSP + 50 Ca | 0.6353 | 0.0142 |
| 3 | Control | PC DSP + 50 Ca | 0.9050 | 0.0007 |

**Killing of *C. albicans* with PC DSP at pH 7.4 with increasing calcium concentrations.**

Three independent experiments were performed. Significant difference (reduction) of mean colony counts was found between control and PC DSP and PC DSP 25 μM calcium (p<0.001). In all other conditions there were no significant **reduction** in mean colony count between mean colony of control and samples with PC DSP and calcium

| **Experiment** | **Treatment1** | **Treatment2** | **Estimate** | **P-value** |
| --- | --- | --- | --- | --- |
| 1 | Control | PC DSP | 6.4206 | <.0001 |
| 1a | Control | PC DSP | 5.9843 | <.0001 |
| 2 | Control | PC DSP | 6.3291 | <.0001 |
| 2a | Control | PC DSP | 6.1179 | <.0001 |
| 3 | Control | PC DSP | 6.2983 | <.0001 |
| 3a | Control | PC DSP | 5.5093 | <.0001 |
| 1a | Control | PC DSP + 100 Ca | 5.9843 | <.0001 |
| 2a | Control | PC DSP + 100 Ca | 4.8883 | <.0001 |
| 3a | Control | PC DSP + 100 Ca | 5.2783 | <.0001 |
| 1a | Control | PC DSP + 200 Ca | 3.5677 | <.0001 |
| 2a | Control | PC DSP + 200 Ca | 3.6157 | <.0001 |
| 3a | Control | PC DSP + 200 Ca | 3.1817 | <.0001 |
| 1 | Control | PC DSP + 25 Ca | 6.4206 | <.0001 |
| 2 | Control | PC DSP + 25 Ca | 6.3291 | <.0001 |
| 3 | Control | PC DSP + 25 Ca | 6.5293 | <.0001 |
| 1 | Control | PC DSP + 50 Ca | 6.4206 | <.0001 |
| 2 | Control | PC DSP + 50 Ca | 6.0980 | <.0001 |
| 3 | Control | PC DSP + 50 Ca | 6.7604 | <.0001 |

**Killing of C. albicans with PC DSP at pH 4 with increasing calcium concentrations.**

Three independent experiments were performed. Significant difference (reduction) of mean colony counts was found between control and all conditions with PC DSP with and without calcium (p<0.001).

).

***Figure*** 5D

| **Experiment** | **Treatment1** | **Treatment2** | **Estimate** | **P-value** |
| --- | --- | --- | --- | --- |
| 1 | Control | LL-37 | 3.2986 | <.0001 |
| 2 | Control | LL-37 | 3.2280 | <.0001 |
| 3 | Control | LL-37 | 4.0296 | <.0001 |
| 1 | Control | LL-37 + 100 Ca | 1.6404 | <.0001 |
| 2 | Control | LL-37 + 100 Ca | 2.5186 | <.0001 |
| 3 | Control | LL-37 + 100 Ca | 2.6194 | <.0001 |
| 1 | Control | LL-37 + 200 Ca | 1.4145 | <.0001 |
| 2 | Control | LL-37 + 200 Ca | 2.7659 | <.0001 |
| 3 | Control | LL-37 + 200 Ca | 3.5282 | <.0001 |
| 1 | Control | LL-37 + 50 Ca | 2.0757 | <.0001 |
| 2 | Control | LL-37 + 50 Ca | 2.0565 | <.0001 |
| 3 | Control | LL-37 + 50 Ca | 2.6878 | <.0001 |
| 1 | Control | LL-37 + 75 Ca | 1.1627 | <.0001 |
| 2 | Control | LL-37 + 75 Ca | 2.6753 | <.0001 |
| 3 | Control | LL-37 + 75 Ca | 2.9703 | <.0001 |

**Killing of *C. albicans* by LL-37 at pH 7.4 with increasing calcium concentrations.**

Two independent experiments were performed. Significant difference (reduction) of mean colony counts was found between control and all conditions with LL-37 with and without calcium (p<0.001).

| **Experiment** | **Treatment1** | **Treatment2** | **Estimate** | **P-value** |
| --- | --- | --- | --- | --- |
| 1 | Control | LL-37 | 1.6339 | <.0001 |
| 2 | Control | LL-37 | 2.4025 | <.0001 |
| 1 | Control | LL-37 + 100 Ca | 1.3942 | <.0001 |
| 2 | Control | LL-37 + 100 Ca | 1.4384 | <.0001 |
| 1 | Control | LL-37 + 200 Ca | 1.3064 | <.0001 |
| 2 | Control | LL-37 + 200 Ca | 1.4253 | <.0001 |
| 1 | Control | LL-37 + 50 Ca | 1.6956 | <.0001 |
| 2 | Control | LL-37 + 50 Ca | 1.8880 | <.0001 |
| 1 | Control | LL-37 + 75 Ca | 1.5909 | <.0001 |
| 2 | Control | LL-37 + 75 Ca | 1.5305 | <.0001 |

**Killing of *C. albicans* by LL-37 at pH 4 with increasing calcium concentrations.**

Two independent experiments were performed. Significant difference (reduction) of mean colony counts was found between control and all conditions with LL-37 with and without calcium (p<0.001).

***Figure 6B***

| **Experiment** | **Treatment1** | **Treatment2** | **Estimate** | **P-value** |
| --- | --- | --- | --- | --- |
| 1 | Control | MSP_66-76EQ_ | 5.3523 | <.0001 |
| 2 | Control | MSP_66-76EQ_ | 5.6354 | <.0001 |
| 3 | Control | MSP_66-76EQ_ | 5.8836 | <.0001 |
| 1 | Control | MSP_66-76EQ_ +Ca | 3.1854 | <.0001 |
| 2 | Control | MSP_66-76EQ_ +Ca | 2.1182 | <.0001 |
| 3 | Control | MSP_66-76EQ_ +Ca | 2.6879 | <.0001 |
| 1 | Control | MSP_66-76EQ_ +Mg | 4.0137 | <.0001 |
| 2 | Control | MSP_66-76EQ_ +Mg | 1.7951 | <.0001 |
| 3 | Control | MSP_66-76EQ_ +Mg | 4.5634 | <.0001 |

**Killing of *C. albicans* by MSP66-76E/Q at pH 4.**

Threee independent experiments were performed. Significant difference (reduction) of mean colony counts was found between control and all conditions with MSP_66-76EQ_ (p<0.001).

***Suppl. figure 1***

***Candida albicans strains***

| **Experiment** | **Treatment1** | **Treatment2** | **Estimate** | **P-value** |
| --- | --- | --- | --- | --- |
| 1 | Control | DSP | -0.08388 | 0.4881 |
| 2 | Control | DSP | -0.2798 | 0.0344 |
| 3 | Control | DSP | 0.3064 | 0.0227 |

**Killing of *C. albicans* SC5814 at pH 7.4**

Three independent experiments were performed. No significant difference (reduction) of mean colony counts was found between control and DSP.

| **Experiment** | **Treatment1** | **Treatment2** | **Estimate** | **P-value** |
| --- | --- | --- | --- | --- |
| 1 | Control | DSP | 4.0551 | <.0001 |
| 2 | Control | DSP | 4.2028 | <.0001 |
| 3 | Control | DSP | 4.7402 | <.0001 |

**Killing of *C. albicans* SC5814 at pH 4**

Three independent experiments were performed. Significant difference (reduction) of mean colony counts was found between control and DSP in all samples (p<0.001).

| **Experiment** | **Treatment1** | **Treatment2** | **Estimate** | **P-value** |
| --- | --- | --- | --- | --- |
| 1 | Control | DSP | 1.0798 | 0.0002 |
| 2 | Control | DSP | 0.5338 | 0.0238 |
| 3 | Control | DSP | 0.1731 | 0.4178 |

**Killing of *C. albicans* BM4f35II68 at pH 7.4**

Three independent experiments were performed. No significant difference (reduction) of mean colony counts was found between control and DSP.

| **Experiment** | **Treatment1** | **Treatment2** | **Estimate** | **P-value** |
| --- | --- | --- | --- | --- |
| 1 | Control | DSP | 3.5349 | <.0001 |
| 2 | Control | DSP | 3.6836 | <.0001 |
| 3 | Control | DSP | 4.0304 | <.0001 |

**Killing of *C. albicans* BM4f35II68 at pH 4**

Three independent experiments were performed. Significant difference (reduction) of mean colony counts was found between control and DSP in all samples (p<0.001).

| **Experiment** | **Treatment1** | **Treatment2** | **Estimate** | **P-value** |
| --- | --- | --- | --- | --- |
| 1 | Control | DSP | -0.3976 | 0.0011 |
| 2 | Control | DSP | 0.01170 | 0.9024 |
| 3 | Control | DSP | -0.1758 | 0.0843 |

**Killing of *C. albicans* CAF 1-2 at pH 7.4**

Three independent experiments were performed. No significant difference (reduction) of mean colony counts was found between control and DSP.

| **Experiment** | **Treatment1** | **Treatment2** | **Estimate** | **P-value** |
| --- | --- | --- | --- | --- |
| 1 | Control | DSP | 4.2280 | <.0001 |
| 2 | Control | DSP | 4.7358 | <.0001 |
| 3 | Control | DSP | 4.3968 | <.0001 |

**Killing of *C. albicans* CAF 1-2 at pH 4**

Three independent experiments were performed. Significant difference (reduction) of mean colony counts was found between control and DSP in all samples (p<0.001).

| **Experiment** | **Treatment1** | **Treatment2** | **Estimate** | **P-value** |
| --- | --- | --- | --- | --- |
| 1 | Control | DSP | 0.8109 | <.0001 |
| 2 | Control | DSP | -0.06297 | 0.6445 |
| 3 | Control | DSP | 0.4050 | 0.0102 |

**Killing of *C. albicans* HLL84 at pH 7.4**

Three independent experiments were performed. No significant difference (reduction) of mean colony counts was found between control and DSP.

| **Experiment** | **Treatment1** | **Treatment2** | **Estimate** | **P-value** |
| --- | --- | --- | --- | --- |
| 1 | Control | DSP | 3.2047 | <.0001 |
| 2 | Control | DSP | 2.8304 | <.0001 |
| 3 | Control | DSP | 3.0532 | <.0001 |

**Killing of *C. albicans* HLL84 at pH 4**

Three independent experiments were performed. Significant difference (reduction) of mean colony counts was found between control and DSP in all samples (p<0.001).

| **Experiment** | **Treatment1** | **Treatment2** | **Estimate** | **P-value** |
| --- | --- | --- | --- | --- |
| 1 | Control | DSP | -1.0679 | 0.0001 |
| 2 | Control | DSP | -0.2591 | 0.1931 |
| 3 | Control | DSP | -0.8512 | 0.0007 |

**Killing of *C. albicans* vaginal clinical isolate SO_303369 at pH 7.4**

Three independent experiments were performed. No significant difference (reduction) of mean colony counts was found between control and DSP.

| **Experiment** | **Treatment1** | **Treatment2** | **Estimate** | **P-value** |
| --- | --- | --- | --- | --- |
| 1 | Control | DSP | 4.4104 | <.0001 |
| 2 | Control | DSP | 4.4230 | <.0001 |
| 3 | Control | DSP | 4.4382 | <.0001 |

**Killing of *C. albicans* vaginal clinical isolate SO_303369 at pH 4**

Three independent experiments were performed. Significant difference (reduction) of mean colony counts was found between control and DSP in all samples (p<0.001).

| **Experiment** | **Treatment1** | **Treatment2** | **Estimate** | **P-value** |
| --- | --- | --- | --- | --- |
| 1 | Control | DSP | -0.08101 | 0.2970 |
| 2 | Control | DSP | 0.03568 | 0.6397 |
| 3 | Control | DSP | -0.05999 | 0.4351 |

**Killing of *C. albicans* vaginal clinical isolate SO_503471 at pH 7.4**

Three independent experiments were performed. No significant difference (reduction) of mean colony counts was found between control and DSP.

| **Experiment** | **Treatment1** | **Treatment2** | **Estimate** | **P-value** |
| --- | --- | --- | --- | --- |
| 1 | Control | DSP | 4.6237 | <.0001 |
| 2 | Control | DSP | 4.6222 | <.0001 |
| 3 | Control | DSP | 4.7359 | <.0001 |

**Killing of *C. albicans* vaginal clinical isolate SO_503471 at pH 4**

Three independent experiments were performed. Significant difference (reduction) of mean colony counts was found between control and DSP in all samples (p<0.001).

***Candida parapsilosis* strains**

| **Experiment** | **Treatment1** | **Treatment2** | **Estimate** | **P-value** |
| --- | --- | --- | --- | --- |
| 1 | Control | DSP | 0.4707 | 0.1205 |
| 2 | Control | DSP | 0.6667 | 0.0356 |
| 3 | Control | DSP | -0.2474 | 0.3969 |

**Killing of *C. parapsilosis* ATC90018 at pH 7.4**

Three independent experiments were performed. No significant difference (reduction) of mean colony counts was found between control and DSP.

| **Experiment** | **Treatment2** | **Treatment2** | **Estimate** | **Probt** |
| --- | --- | --- | --- | --- |
| 1 | Control | DSP | 4.0645 | <.0001 |
| 2 | Control | DSP | 4.3774 | <.0001 |
| 3 | Control | DSP | 4.1437 | <.0001 |

**Killing of *C. parapsilosis* ATC90018 at pH 4**

Three independent experiments were performed. Significant difference (reduction) of mean colony counts was found between control and DSP in all samples (p<0.001).

| **Experiment** | **Treatment1** | **Treatment2** | **Estimate** | **P-value** |
| --- | --- | --- | --- | --- |
| 1 | Control | DSP | 0.2634 | 0.0076 |
| 2 | Control | DSP | 0.1664 | 0.0658 |
| 3 | Control | DSP | -0.6716 | <.0001 |

**Killing of *C. parapsilosis* BD17837 at pH 7.4**

Three independent experiments were performed. No significant difference (reduction) of mean colony counts was found between control and DSP.

| **Experiment** | **Treatment1** | **Treatment2** | **Estimate** | **P-value** |
| --- | --- | --- | --- | --- |
| 1 | Control | DSP | 3.5102 | <.0001 |
| 2 | Control | DSP | 3.9888 | <.0001 |
| 3 | Control | DSP | 3.5391 | <.0001 |

**Killing of *C. parapsilosis* BD17837 at pH 4**

Three independent experiments were performed. Significant difference (reduction) of mean colony counts was found between control and DSP in all samples (p<0.001).

| **Experiment** | **Treatment1** | **Treatment2** | **Estimate** | **P-value** |
| --- | --- | --- | --- | --- |
| 1 | Control | DSP | 0.8775 | <.0001 |
| 2 | Control | DSP | 0.5216 | 0.0004 |
| 3 | Control | DSP | -0.4908 | 0.0006 |

**Killing of *C. parapsilosis* BD18800 at pH 7.4**

Three independent experiments were performed. No significant difference (reduction) of mean colony counts was found between control and DSP (both reduction and increase is seen).

| **Experiment** | **Treatment1** | **Treatment2** | **Estimate** | **P-value** |
| --- | --- | --- | --- | --- |
| 1 | Control | DSP | 4.2158 | <.0001 |
| 2 | Control | DSP | 4.2147 | <.0001 |
| 3 | Control | DSP | 2.7532 | <.0001 |

**Killing of *C. parapsilosis* BD18800 at pH 4**

Three independent experiments were performed. Significant difference (reduction) of mean colony counts was found between control and DSP in all samples (p<0.001).

| **Experiment** | **Treatment1** | **Treatment2** | **Estimate** | **P-value** |
| --- | --- | --- | --- | --- |
| 1 | Control | DSP | -0.2013 | 0.2857 |
| 2 | Control | DSP | 0.6227 | 0.0047 |
| 3 | Control | DSP | -0.4845 | 0.0197 |

**Killing of *C. parapsilosis* BM2468/09 at pH 7.4**

Three independent experiments were performed. No significant difference (reduction) of mean colony counts was found between control and DSP (both reduction and increase is seen).

| **Experiment** | **Treatment1** | **Treatment2** | **Estimate** | **P-value** |
| --- | --- | --- | --- | --- |
| 1 | Control | DSP | 2.8470 | <.0001 |
| 2 | Control | DSP | 3.0904 | <.0001 |
| 3 | Control | DSP | 3.7698 | <.0001 |

**Killing of *C. parapsilosis* BM2468/09 at pH 4**

Three independent experiments were performed. Significant difference (reduction) of mean colony counts was found between control and DSP in all samples (p<0.001).

***Candida glabrata* strains**

| **Experiment** | **Treatment1** | **Treatment2** | **Estimate** | **P-value** |
| --- | --- | --- | --- | --- |
| 1 | Control | DSP | -0.3068 | 0.0366 |
| 2 | Control | DSP | -0.09622 | 0.4749 |
| 3 | Control | DSP | -0.9025 | <.0001 |

**Killing of *C. glabrata* ATC 90030 at pH 7.4**

Three independent experiments were performed. No significant difference of mean colony counts was found between control and DSP.

| **Experiment** | **Treatment1** | **Treatment2** | **Estimate** | **P-value** |
| --- | --- | --- | --- | --- |
| 1 | Control | DSP | -0.8270 | 0.0006 |
| 2 | Control | DSP | -2.0924 | <.0001 |
| 3 | Control | DSP | -1.4733 | <.0001 |

**Killing of *C. glabrata* ATC 90030 at pH 4**

Three independent experiments were performed. Significant difference of mean colony counts was found between control and DSP in all samples (p<0.001) (treatment with DSP increased the mean colony count).

| **Experiment** | **Treatment1** | **Treatment2** | **Estimate** | **P-value** |
| --- | --- | --- | --- | --- |
| 1 | Control | DSP | -1.1610 | <.0001 |
| 2 | Control | DSP | -1.0600 | <.0001 |
| 3 | Control | DSP | -0.7964 | <.0001 |

**Killing of *C. glabrata* clinical isolate SO_3400 at pH 7.4**

Three independent experiments were performed. Significant difference of mean colony counts was found between control and DSP in all samples (p<0.001) (treatment with DSP increased the mean colony count).

| **Experiment** | **Treatment1** | **Treatment2** | **Estimate** | **P-value** |
| --- | --- | --- | --- | --- |
| 1 | Control | DSP | -1.3035 | <.0001 |
| 2 | Control | DSP | -0.5795 | 0.0001 |
| 3 | Control | DSP | -0.8331 | <.0001 |

**Killing of *C. glabrata* clinical isolate SO_3400 at pH 4**

Three independent experiments were performed. Significant difference of mean colony counts was found between control and DSP in all samples (p<0.001) (treatment with DSP increased the mean colony count).

| **Experiment** | **Treatment1** | **Treatment2** | **Estimate** | **P-value** |
| --- | --- | --- | --- | --- |
| 1 | Control | DSP | 0.005538 | 0.9666 |
| 2 | Control | DSP | -1.1007 | <.0001 |
| 3 | Control | DSP | -0.8257 | <.0001 |

**Killing of *C. glabrata* clinical isolate SO_3336 at pH 7.4**

Three independent experiments were performed. No significant difference of mean colony counts was found between control and DSP.

| **Experiment** | **Treatment1** | **Treatment2** | **Estimate** | **P-value** |
| --- | --- | --- | --- | --- |
| 1 | Control | DSP | -0.7196 | 0.0001 |
| 2 | Control | DSP | 0.4636 | 0.0043 |
| 3 | Control | DSP | 0.2446 | 0.0891 |

**Killing of *C. glabrata* clinical isolate SO_3336 at pH 4**

Three independent experiments were performed. No significant difference of mean colony counts was found between control and DSP.

| **Experiment** | **Treatment1** | **Treatment2** | **Estimate** | **P-value** |
| --- | --- | --- | --- | --- |
| 1 | Control | DSP | 0.2004 | 0.0717 |
| 2 | Control | DSP | -0.06014 | 0.5643 |
| 3 | Control | DSP | -0.3646 | 0.0037 |

**Killing of *C. glabrata* clinical isolate SO_503413 at pH 7.4**

Three independent experiments were performed. No significant difference of mean colony counts was found between control and DSP.

| **Experiment** | **Treatment1** | **Treatment2** | **Estimate** | **P-value** |
| --- | --- | --- | --- | --- |
| 1 | Control | DSP | -0.5420 | 0.2659 |
| 2 | Control | DSP | -0.04437 | 0.9255 |
| 3 | Control | DSP | 0.8320 | 0.0984 |

**Killing of *C. glabrata* clinical isolate SO_503413 at pH 4**

Three independent experiments were performed. No significant difference of mean colony counts was found between control and DSP.

***Suppl. figure 4***

| **Experiment** | **Treatment1** | **Treatment2** | **Estimate** | **P-value** |
| --- | --- | --- | --- | --- |
| 1 | Control | PMSP | 3.2153 | <.0001 |
| 2 | Control | PMSP | 1.7637 | <.0001 |
| 3 | Control | PMSP | 5.2034 | <.0001 |
| 1 | Control | PMSP+EDTA | 4.1924 | <.0001 |
| 2 | Control | PMSP+EDTA | 5.0730 | <.0001 |
| 3 | Control | PMSP+EDTA | 5.0331 | <.0001 |

**Killing of *C. albicans* with PMSP at pH 4.**

Threee independent experiments were performed. Significant difference (reduction) of mean colony counts was found between control and all conditions with PMSP with and without EDTA (p<0.001).
