# Supplementary figures and images for: β-Microseminoprotein Endows Post Coital Seminal Plasma with Potent Candidacidal Activity by a Calcium- and pH-Dependent Mechanism
Source: PLoS Pathog. 2012 Apr 5;8(4):e1002625. doi: 10.1371/journal.ppat.1002625 (PMC3320615; doi:10.1371/journal.ppat.1002625)

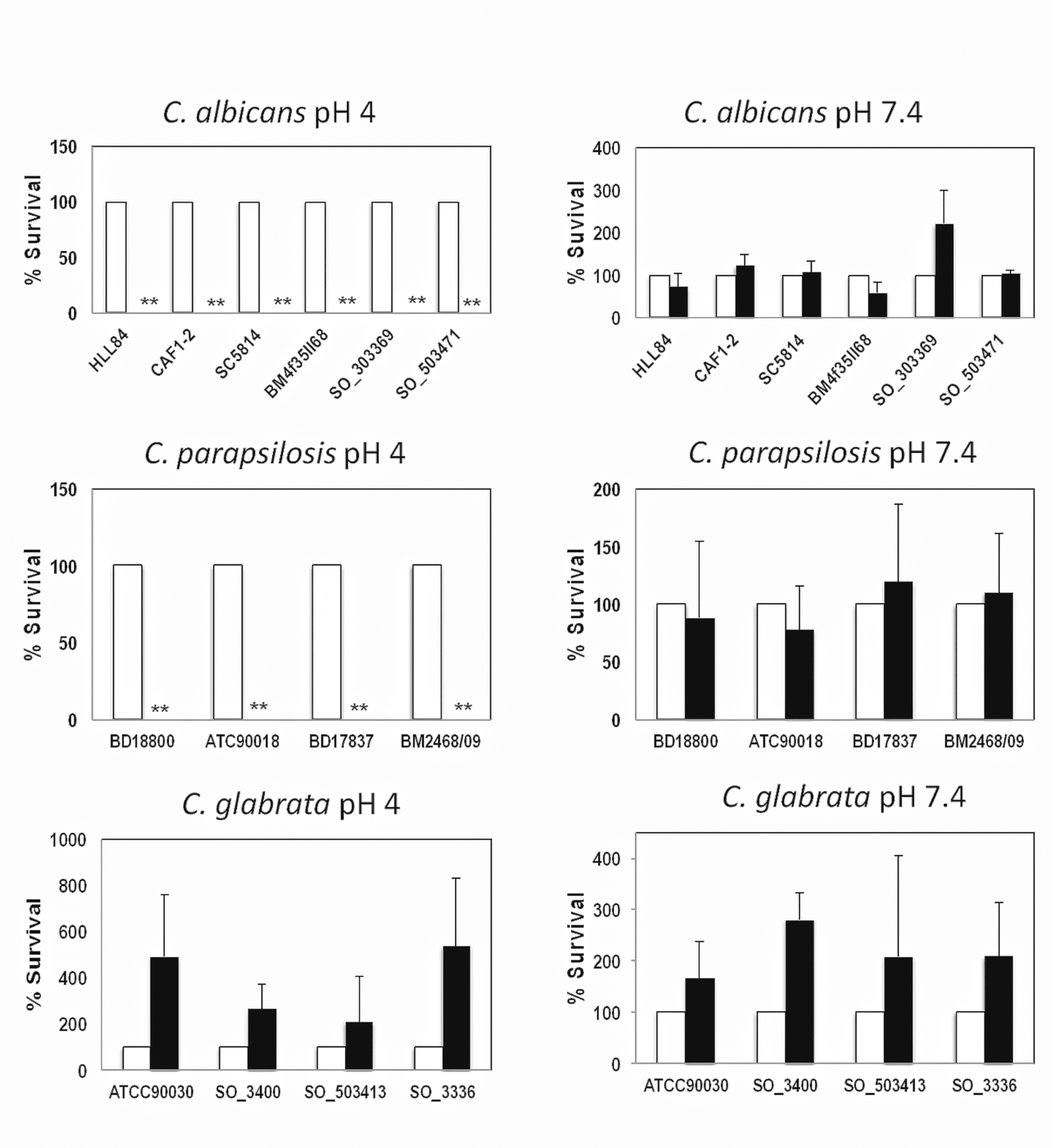

Supplement: Figure S1 — Activity of dialyzed seminal plasma against Candida strains. The candidacidal activity of 1/50 dilution of dialyzed seminal plasma (2%) was tested against strains of C. albicans, C. parapsilosis, and C. glabrata. At pH 4, more than 10 colonies were found in 1000-fold dilution of the control samples, but no colonies were found in 10-fold dilution of the samples of C. albicans and C. parapsilosis treated with dialyzed seminal plasma corresponding to a more than 3 log reduction. In contrast, no significant killing was observed at pH 7 or in any of the C. glabrata samples. All results are shown as the average from three independent experiments. ** denotes p<0.001. Error bars indicate standard deviations. Further details regarding the statistical analysis are found in Dataset S1. (Black bars: dialyzed seminal plasma, white bars: control). (TIF) [file ppat.1002625.s002.tif]

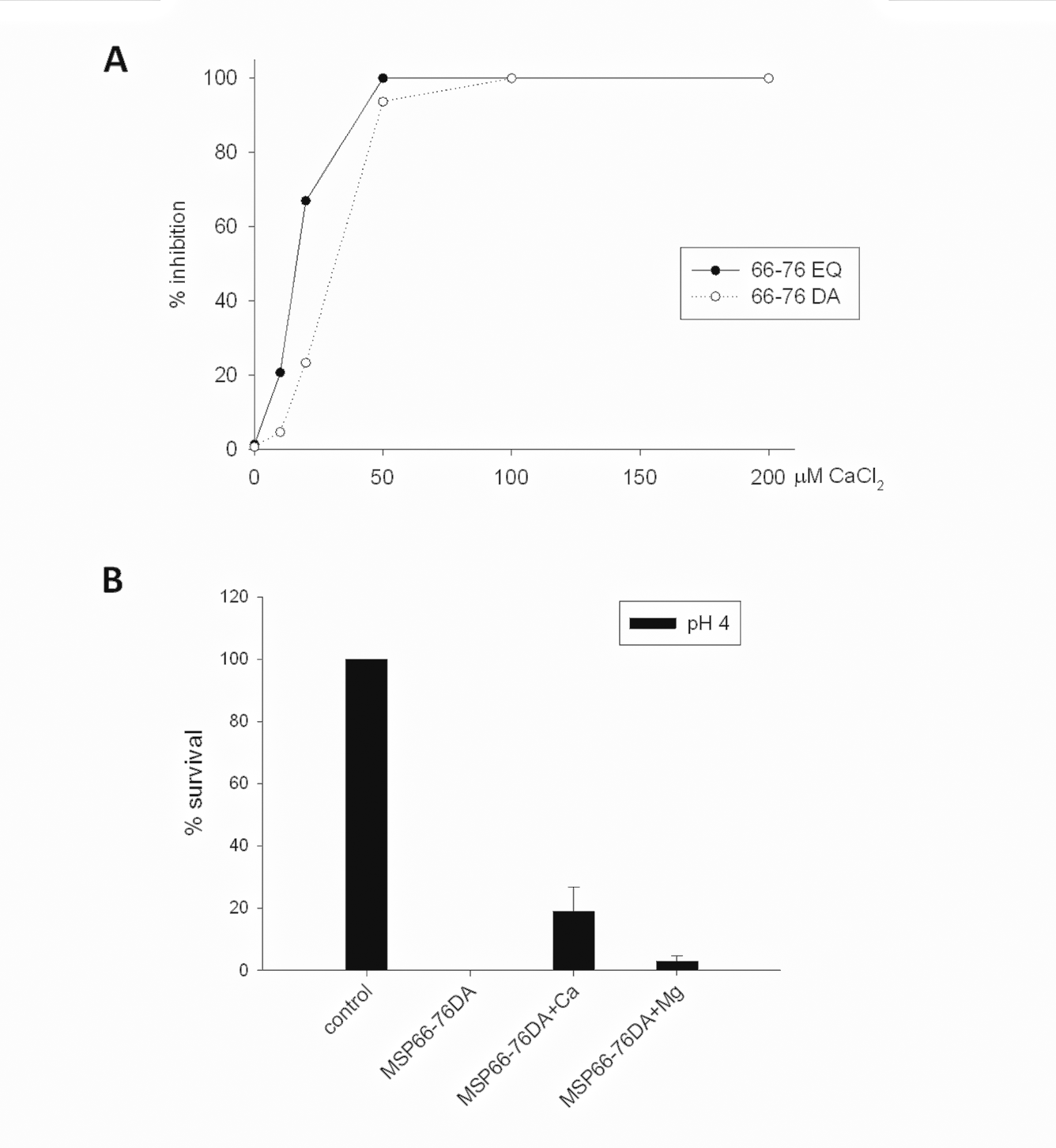

Supplement: Figure S2 — Candidacidal activities of MSP66–76E/Q and MSP66–76D/A. A. While the antifungal activity of MSP66–76E/Q was not inhibited by calcium (0.5 mM) at acidic pH, the activity was still inhibited at 50–100 µM calcium at neutral pH. However, even at neutral pH the activity was more calcium resistant than MSP66–76 since this peptide only was active at neutral pH with equimolar concentration of EDTA. Similar inhibition by calcium at neutral pH was found for MSP66–76D/A where the aspartic acid (D) adjacent to the glutamic acid was substituted with an alanine (A). B. The antifungal activity of MSP66–76D/A was not inhibited by even 0.5 mM calcium at acidic pH. Coordination of calcium in proteins typically involves six different atoms normally originating from up to six different residues and including backbone carbonyls. It is therefore not surprising that single or even double mutation in the peptide preserved calcium binding, but with an expected lower affinity. (TIF) [file ppat.1002625.s003.tif]

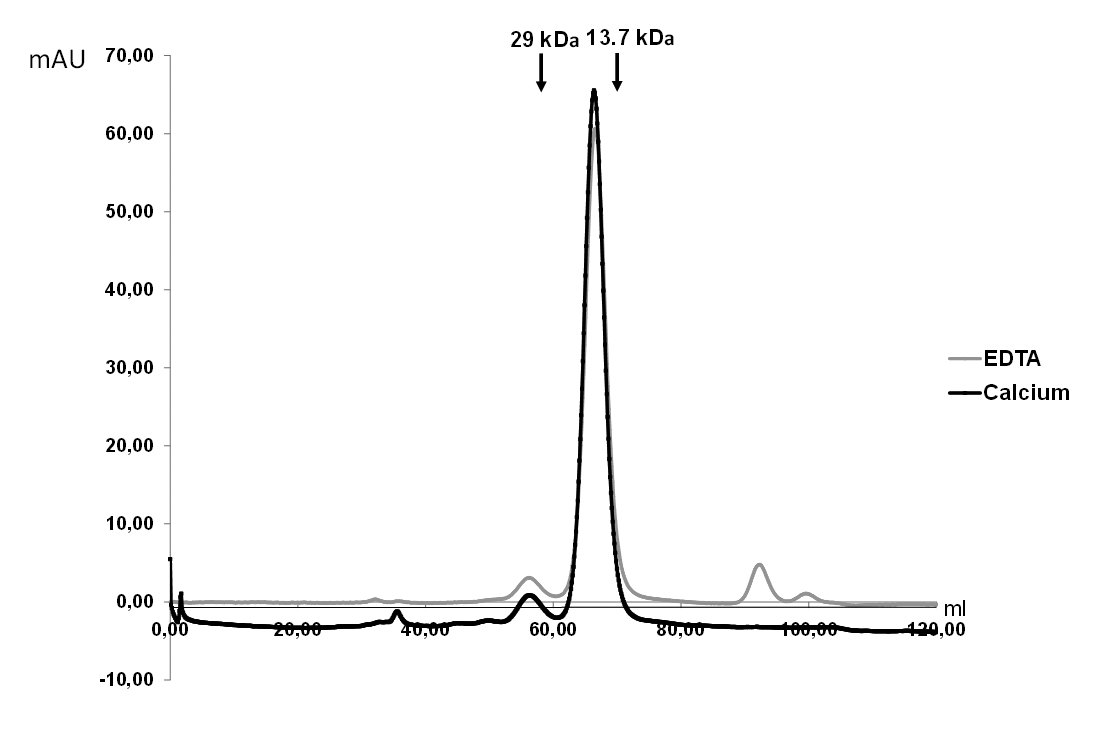

Supplement: Figure S3 — Seize exclusion chromatography of MSP. Native MSP was analyzed by seize exclusion chromatography on a HiPrep 16/60 Sephacryl S 100 column in the presence of 1 mM calcium or 1 mM EDTA in Trisbuffer (50 mM Tris, 150 mM NaCl, pH 7.5). (TIF) [file ppat.1002625.s004.tif]

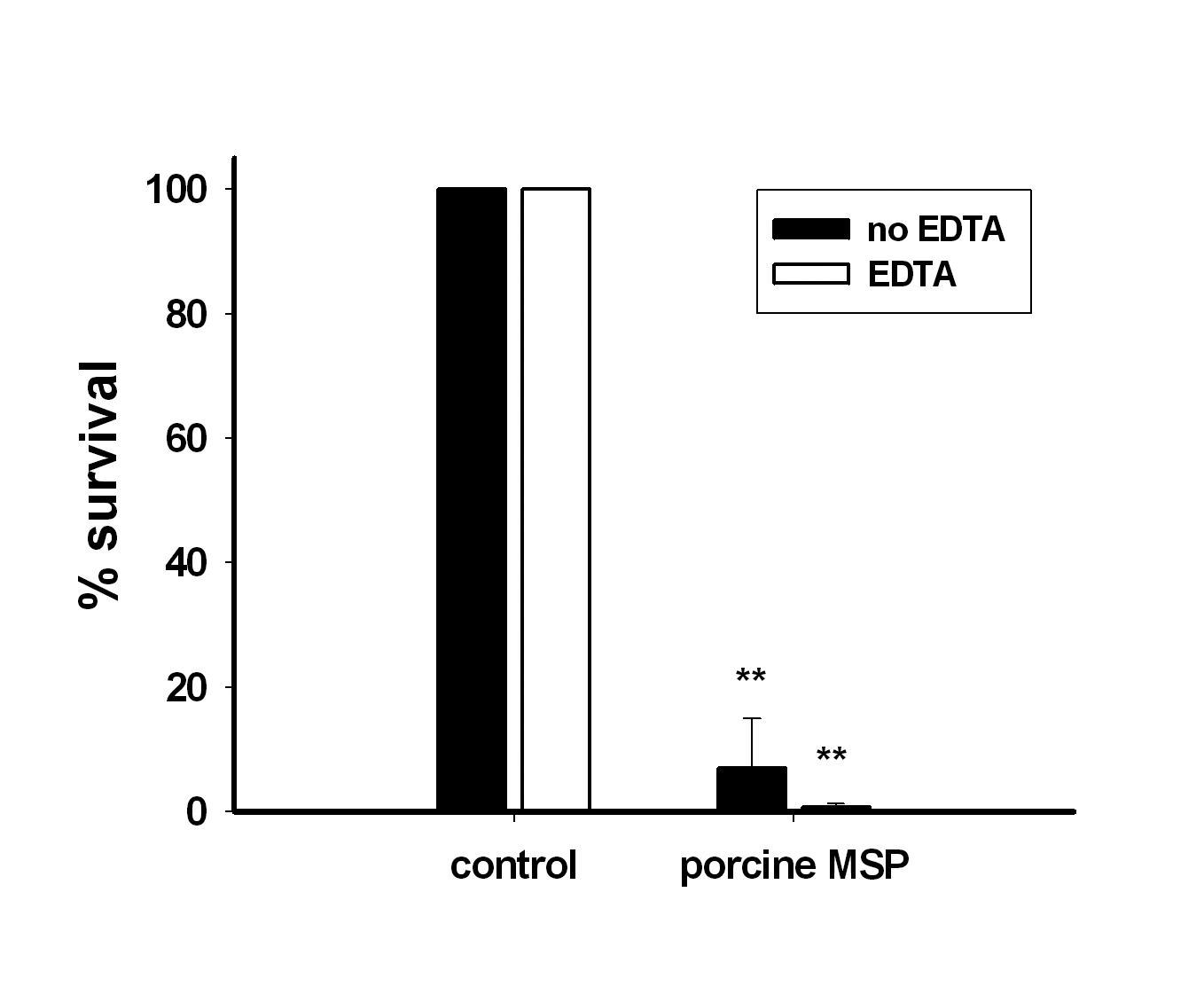

Supplement: Figure S4 — Antifungal activity of peptide derived from porcine MSP. Porcine MSP was tested in CFU assay at pH 4 with and without EDTA. All results are shown as the average from three independent experiments. ** denotes p<0.001. Error bars indicate standard deviations. Further details regarding the statistical analysis are found in Dataset S1. (TIF) [file ppat.1002625.s005.tif]

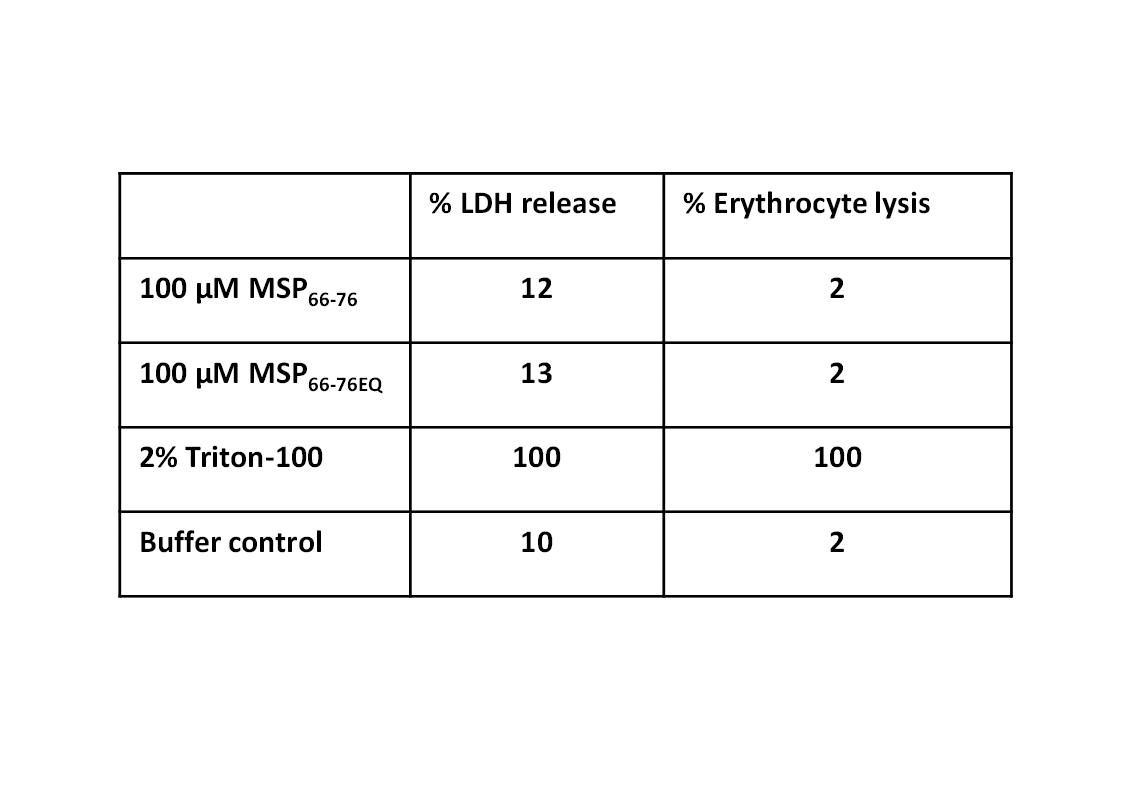

Supplement: Table S1 — Cytoxic and hemolytic Effect of MSP66–76E/Q and MSP66–76. HaCaT cells were treated with 100 µM of MSP66–76E/Q and MSP66–76 over night. The release of LDH was subsequently determined by colorimetric assay and depicted as percent of LDH release of cells treated with 2% Triton- X100. Erythrocytes were treated for 1 ½ hour with 100 µM of MSP66–76E/Q and MSP66–76 followed by centrifugation. Lysis was determined by measuring hemoglobin in the supernatant by measuring the absorbance at 550 nm. Results are depicted as % of lysis compared to lysis obtained by 2% Triton-X100. (TIF) [file ppat.1002625.s006.tif]
